# A Novel Van der Woude Syndrome-Causing *IRF6* Variant Is Subject to Incomplete Non-sense-Mediated mRNA Decay Affecting the Phenotype of Keratinocytes

**DOI:** 10.3389/fcell.2020.583115

**Published:** 2020-09-29

**Authors:** Martin Degen, Eleftheria Girousi, Julia Feldmann, Ludovica Parisi, Giorgio C. La Scala, Isabelle Schnyder, André Schaller, Christos Katsaros

**Affiliations:** ^1^Laboratory for Oral Molecular Biology, Dental Research Center, Department of Orthodontics and Dentofacial Orthopedics, University of Bern, Bern, Switzerland; ^2^Division of Pediatric Surgery, Department of Pediatrics, University Hospital of Geneva, Geneva, Switzerland; ^3^University Clinic for Pediatric Surgery, Bern University Hospital, Bern, Switzerland; ^4^Division of Human Genetics, Bern University Hospital, Bern, Switzerland

**Keywords:** Van der Woude syndrome, IRF6, cleft lip/palate, non-sense-mediated mRNA decay, haploinsuffiency, epidermal differentiation

## Abstract

Van der Woude syndrome (VWS) is a genetic syndrome that leads to typical phenotypic traits, including lower lip pits and cleft lip/palate (CLP). The majority of VWS-affected patients harbor a pathogenic variant in the gene encoding for the transcription factor interferon regulatory factor 6 (IRF6), a crucial regulator of orofacial development, epidermal differentiation and tissue repair. However, most of the underlying mechanisms leading from pathogenic *IRF6* gene variants to phenotypes observed in VWS remain poorly understood and elusive. The availability of one VWS individual within our cohort of CLP patients allowed us to identify a novel VWS-causing *IRF6* variant and to functionally characterize it. Using VWS patient-derived keratinocytes, we reveal that most of the mutated *IRF6_VWS* transcripts are subject to a non-sense-mediated mRNA decay mechanism, resulting in IRF6 haploinsufficiency. While moderate levels of *IRF6_VWS* remain detectable in the VWS keratinocytes, our data illustrate that the IRF6_VWS protein, which lacks part of its protein-binding domain and its whole C-terminus, is noticeably less stable than its wild-type counterpart. Still, it maintains transcription factor function. As we report and characterize a so far undescribed VWS-causing *IRF6* variant, our results shed light on the physiological as well as pathological role of IRF6 in keratinocytes. This acquired knowledge is essential for a better understanding of the molecular mechanisms leading to VWS and CLP.

## Introduction

Craniofacial morphogenesis represents one of the most intricate developmental processes of our body. It involves several spatiotemporally regulated gene regulatory networks (GRN) that control cellular mechanisms, such as differentiation, migration, proliferation, and apoptosis, driving outgrowth, patterning, and fusion of facial structures. This immense complexity makes the formation of the craniofacial components highly susceptible to dysregulations that can lead to anomalies, such as cleft lip/palate (CLP) (Wilkie and Morriss-Kay, 2001).

CLP is a deformity resulting from failure of the facial processes to grow or fuse properly during embryogenesis (Deshpande and Goudy, 2019). With an average incidence rate of one in 750 newborns, CLP belongs to the most frequent human birth anomalies worldwide (Kousa and Schutte, 2016). Although this malformation is not a major cause of mortality in developed countries, it still poses an enormous psychological, social, and economic burden for cleft-affected patients, their families, and society as a whole (Wehby and Cassell, 2010). Furthermore, even after the need for the young CLP patients to endure several corrective surgeries, they may still experience complications and consequences of their anomalies, including problems with speech, hearing, wound healing, scar and tissue contractures, and facial asymmetries (Van Beurden et al., 2005; Hortis-Dzierzbicka et al., 2012).

CLP is etiologically very complex and heterogeneous, involving a combination of genetic and environmental risk factors, and it can occur either in syndromic or non-syndromic form (Mossey et al., 2009). While non-syndromic CLP represents the majority of cases and lacks any phenotypic features outside of the orofacial region, syndromic individuals present with additional anomalies outside of the clefting area. To date, numerous genetic risk factors have been identified and associated with orofacial clefting (Gritli-Linde, 2007; Juriloff and Harris, 2008; Dixon et al., 2011; Bush and Jiang, 2012). However, the individual cause and the underlying mechanism is only known for the minority of CLP cases (Meng et al., 2009).

Van der Woude syndrome (VWS; OMIM # 119300) is one of the most common syndromic forms of orofacial clefts, accounting for 2% of all CLP cases (Rizos and Spyropoulos, 2004; Little et al., 2009). VWS is inherited as an autosomal dominant condition with high penetrance and variable expressivity (Peyrard-Janvid et al., 2005) associated with one or more of the following characteristics: lower lip pits/fistulae, CLP, or submucous cleft palate (Van Der Woude, 1954). Recent studies have further identified a higher risk of wound healing complications following surgical cleft repair and a thicker epidermis with more proliferating keratinocytes in VWS patients when compared to non-syndromic children (Jones et al., 2010; Hixon et al., 2016). Hence, GRNs seem to be involved in the pathogenesis of VWS that orchestrate craniofacial morphogenesis, tissue repair, and epidermal differentiation. So far, three genes have been causatively linked to VWS: (1) Loss-of-function mutations in *Interferon Regulatory Factor 6* (*IRF6*) are responsible for 72% of VWS cases, but pathogenic *IRF6* variants are also found in the more severe Popliteal Pterygium syndrome (PPS; OMIM # 119500) (Kondo et al., 2002) and in non-syndromic CLP (Zucchero et al., 2004); (2) dominant-negative variants of *Grainyhead-like transcription factor* 3 (*GRHL3*) have been reported in 5% of VWS patients not having *IRF6* mutations (Peyrard-Janvid et al., 2014); (3) a rare pathogenic missense variant in *NME/NM23 Nucleoside Diphosphate Kinase 1* (*NME1*) has been recently identified in *IRF6* and *GRHL3* mutation-negative VWS individuals (Parada-Sanchez et al., 2017).

IRF6 is a transcription factor regulating the balance between immature ectoderm differentiation and proliferation during craniofacial morphogenesis. *Irf6*-deficient mice display a thickened and disorganized oral epithelium, reduced skin barrier function, and premature aberrant oral adhesions, resulting in orofacial clefts (Ingraham et al., 2006; Richardson et al., 2006). While IRF6-truncating heterozygous variants are often found in VWS patients (de Lima et al., 2009), detailed descriptions of genotype-phenotype correlations in VWS are still missing.

Here, we describe a novel heterozygous VWS-causing *IRF6* frameshift variant c.961_965delGTGTAinsC/p.(Val321Profs^∗^15) affecting the highly conserved protein-binding domain SMIR/IAD of IRF6 [SMAD/IRF (SMIR) or interferon association domain (IAD) (Eroshkin and Mushegian, 1999)]. Using VWS and non-syndromic CLP patient-derived keratinocytes, we show that the *IRF6_VWS* transcript undergoes an incomplete non-sense-mediated RNA decay (NMD) process leading to IRF6 haploinsufficiency. Additionally, we reveal that the IRF6_VWS protein is less stable than its wild-type (wt) counterpart, but that it maintains its function as a transcription factor. Using primary patient cells as well as over-expression systems, we present a thorough functional characterization of a novel VWS-causing *IRF6* variant and elucidate how the specific variant affects the phenotype of keratinocytes.

## Materials and Methods

### Ethics Statement

This work was performed according to the Ethical Principles for Medical Research Involving Human Subjects as defined by the World Medical Association (WMA Declaration of Helsinki - Ethical principles for medical research involving human subjects. Available at: https://www.wma.net/policies-post/wma-declaration-of-helsinki-ethical-principles-for-medical-research-involving-human-subjects). Isolation of human cleft lip- as well as control tissue-derived keratinocytes and their analyses for this study have been approved by the Kantonale Ethikkommission of Bern, Switzerland (protocol number: 2017-01394). Informed written consent was obtained from the patient’s parents.

### Cell Culture

Cleft lip tissue samples were obtained from three to seven months old patients undergoing primary corrective surgery at the Children’s Hospital, University of Bern. Clinical assessment and surgeries were all performed by two highly experienced clinicians (I.S. and G.C. LS). In addition, we included three healthy control biopsies: lip tissue from a four-year-old child sustaining a lip laceration; foreskin from a two-year-old boy undergoing circumcision; forehead skin from an 18-month-old infant experiencing a traumatic incident.

Patient-derived cells were isolated from the tissue as described previously using the explant culture system (Degen et al., 2018). After their isolation, keratinocytes were cultured in keratinocyte serum-free medium (KSFM, Gibco, Thermo Fisher Scientific, Lucerne, Switzerland) supplemented with 25 μg/ml bovine pituitary extract, 0.2 ng/ml epidermal growth factor (EGF), and CaCl_2_ to a final Ca^2+^ concentration of 0.4 mM, as described elsewhere (Degen et al., 2012). To maintain healthy cells, keratinocyte cultures reaching 40% confluency were re-fed daily with 1:1 medium (1:1 vol/vol Ca^2+^-free Dulbecco’s modified Eagle’s medium (DMEM) with complete KSFM). Experiments using primary cells were all performed with cultures from the second to fourth passage. Each of the keratinocyte primary cell cultures originating from individual donors represents a mixture of cells that grew out of multiple explants. All cells were tested for mycoplasma contamination prior to freezing.

The immortalized oral mucosal keratinocytes OKF6/TERT2 were grown in KSFM as described elsewhere (Dickson et al., 2000). The commercially available human keratinocyte cell line HaCaT (#300493) and the human embryonic kidney cell line HEK293 (#300192; both from CLS cell line service, Eppelheim, Germany) were cultured in DMEM containing 10% fetal calf serum (FCS, Sigma-Aldrich, St. Louis, MO, United States) and 1 x Pen/Strep solution (Gibco, Thermo Fisher Scientific).

Details and characteristics of all the cells used in this study are summarized in Table 1.

**TABLE 1:** Cells that have been used in this study, including cell name, donor sex, donor age, and characteristics are indicated.

**Cells**	**Donor sex**	**Donor age**	**Tissue**	**Specifics**	**Syndrome**	**VWS-linked mutation in *IRF6*, *GRHL3*, *NME1***	
**Primary keratinocytes**							
VWS (D6)	M	16 weeks	Lip	Bilateral complete CLP	VWS	*IRF6* (c.961delGTGTAinsC)	VWS
CLP1 (E6)	M	15 weeks	Lip	Partial cleft lip left, cleft palate	no*	no	
CLP2 (F6)	F	21 weeks	Lip	Complete CLP left	no*	no	Non-syndromic CLP
CLP3 (PA)	M	30 weeks	Lip	Incomplete cleft lip (left)	no*	no	
CLP4 (H7)	M	14 weeks	Lip	Complete CLP right	no*	no	
Lip (19K)	n.a.	4 years	Lip	Healthy	no	not applicable	
Forehead (19H)	n.a.	1.5 years	Forehead	Healthy	no	not applicable	Control
Foreskin (18E)	M	2 years	Foreskin	Healthy	no	not applicable	

**Cells**	**Donor sex**	**Donor age**	**Tissue**	**Specifics**			

**Other cells**							
OKF6/TERT2	M	57 years	Floor of the mouth	Healthy; oral mucosal keratinocytes			
HaCaT	M	62 years	Skin	Healthy; *in vitro* spontaneously transformed keratinocytes			
HEK293		Fetus	Kidney	Healthy			

M: male. F: female. n.a. not available. * verified by whole exome sequencing.

### Immunofluorescence

For cell staining, cells were grown in 35 mm dishes containing four separate wells (Greiner Bio-One, Frickenhausen, Germany). Cells were washed twice with phosphate-buffered saline (PBS) before fixation in 4% paraformaldehyde (PFA) for 15 min at room temperature. Afterward, fixed cells were washed three times in PBS, permeabilized in 0.1% Triton-X-100 for 5 min and incubated with primary antibody for 2 h at room temperature. After three PBS rinses, cells were incubated, light-protected, with fluorescent-labeled secondary antibodies (Molecular Probes, Thermo Fisher Scientific) and/or tetramethylrhodamine (TRITC)-phalloidin (Sigma-Aldrich) for 1 h, rinsed with PBS and coverslip-mounted with Vectashield Mounting Medium containing DAPI (Vector Laboratories, Burlingame, CA, United States). Cells were examined under an Olympus BX-51 phase/fluorescence microscope (Olympus Life Science Solutions Tokyo, Japan) equipped with a xenon lamp (X-Cite, series 120PC Q, Lumen Dynamics, Mississauga, Canada), and fluorescence filters U-MWIBA3 for AlexaFluor 488 and U-MNUA2 for DAPI (Olympus Life Science Solutions). Images were captured by a ProgRes CT3 camera with ProgRes CapturePro software (Jenaoptik, Jena, Germany), using a 20x/0.5 objective. Primary antibodies used: anti-myc (clone 9E10, Abcam, Cambridge, United Kingdom), anti-IRF6 (clone 14B2C16, BioLegend, San Diego, CA, United States), and anti-E-Cadherin (20874-1-AP, Proteintech, Manchester, United Kingdom).

For IRF6 localization and quantification, linescan plots were performed using the ImageJ software version 1.50f^1^ (NIH, Bethesda, MD, United States). Briefly, a line, spanning an entire cell, was drawn and the gray intensities along the line (through the cell) were plotted for the DAPI (blue) as well as for the IRF6 channel (green).

### 3D-Cultures

To generate 3D-skin models using CLP patient-derived keratinocytes, the protocol from CELLnTEC (CELLnTEC advanced cells systems AG, Bern, Switzerland) was used. Briefly, 2 × 10^5^ cells were seeded in 400 μl KSFM into polycarbonate inserts (0.4 μm pore size, 12 mm diameter, Nunc, Thermo Fisher Scientific) placed in 60 mm tissue culture dishes, immediately followed by the addition of 11 ml of KSFM outside the inserts. To confirm confluency, one insert from each culture was stained two days after seeding with the staining kit (CnT-ST-100, CELLnTEC) and observed with brightfield microscopy. If the monolayer was confluent, keratinocyte differentiation was induced in the parallel culture by the replacement of KSFM with 3D Barrier Medium (CnT-PR-3D, CELLnTEC) inside and outside of the insert (equal medium level) overnight. The insert was then lifted to the air-liquid interface by carefully removing the medium inside the insert and replenishing the outside medium with fresh 3D Barrier Medium up to the level of the membrane (approximately 3.2 ml for a p60 culture dish). Keratinocytes were cultured for 15 days at 37°C and 5% CO_2_ with medium change every other day.

To analyze the skin models, 3D-cultures were fixed overnight at 4°C in 4% PFA. Membranes were cut out of the inserts, placed between two biopsy pads in embedding cassettes, and stored in 0.1 M sodium cacodylate buffer at 4°C before proceeding for paraffin embedding and sectioning on a Reichert-Jung microtome (Leica Microsystems, Heerbrugg, Switzerland). Slides containing 5 μm thick paraffin sections were deparaffinized and rehydrated through xylene, ethanol, and deionized H_2_O, stained with hematoxylin and eosin (H&E) or anti-Loricrin antibody (PA5-30583, Thermo Fisher Scientific). Immunostainings and microscopy were performed as described above for the cells. For the quantification of Loricrin-positive areas the ImageJ software was used.

### Next Generation Sequencing (NGS)

For NGS, VWS patient DNA was extracted from EDTA-blood using the Prepito DNA Blood600 Kit (PerkinElmer, Waltham, MA, United States). Targeted exome capture sequencing was performed using the TruSight One sequencing Kit on a MiSeq Sequencing system (Illumina, San Diego, CA, United States) according to the manufacturer’s instructions. Sequencing data were analyzed using CLC Workbench (Qiagen, Hilden, Germany) using GRCh37hg19 as reference genome. The candidate variant was verified by Sanger sequencing.

Pathogenic variants in the known VWS-associated genes *IRF6* (NM_006147.4), *GRHL3* (NM_198173), and *NME1* (NM_198175) have been excluded in the CLP patient-derived cells CLP1-CLP4 by whole exome sequencing (WES) using the Twist human core exome Kit including RefSeq spike in (TWIST Biosciences, San Francisco, CA, United States) on a NextSeq 500 instrument (Illumina).

### RNA Extraction, cDNA Synthesis, and Quantitative Real-Time PCR (qPCR) Analysis

Total RNA was isolated from cells using the innuPREP RNA Mini kit (Analytik Jena AG, Jena, Germany) according to their standard protocol for eukaryotic cells. RNA quality and concentration were assessed using a Nanodrop 2000c instrument (Thermo Fisher Scientific). RNA was stored at −80°C until further use.

First strand cDNA was synthesized from 500 ng total RNA using the M-MLV Reverse Transcriptase (Promega, Dübendorf, Switzerland) and Oligo(dT)_15_ Primer (Promega). mRNA levels were analyzed and quantified by qPCR using GoTaq^®^ qPCR Master Mix (Promega) on a QuantStudio 3 instrument (Applied Biosystems, Thermo Fisher Scientific). Relative RNA expression was calculated using the ΔΔC_T_ method, normalizing values to *GAPDH* within each sample.

Sequences of the qPCR primers (Supplementary Table S1) were either taken from the PrimerBank database^2^ or from the NCBI primer designing tool^3^. All qPCR primer pairs were tested for specificity and efficiency using cDNA standard curves.

### Non-sense-mediated mRNA Decay (NMD) Analysis

VWS-, non-syndromic CLP (CLP1), and healthy control lip-derived keratinocytes (lip) were cultured either in KSFM or in KSFM including the NMD inhibitors Puromycin for 4 h (100 μg/ml, Sigma-Aldrich) or cycloheximide for 4 h (CHX, 100 μg/ml, Sigma-Aldrich). RNAs were isolated from both cell lines and conditions and cDNAs synthesized. cDNAs were used as templates for qPCR analyses using specific primers recognizing *IRF6_VWS*, total *IRF6*, and *IRF6wt*, respectively. In addition, VWS- and CLP1- derived cDNAs were used as templates for PCR amplifications of a 389 bp IRF6 fragment (spanning exon 7–9) encompassing the specific *IRF6_VWS* variant at position 961 base pairs (bp) using the primers 5′-GAGCAGGTCAAATTCCCAGG-3′/5′-ATCCGAGCCACTACTGGAATGAC-3′. PCR amplicons were run on an agarose gel, DNA extracted, purified, and sequenced using the primers mentioned above. Chromatograms were analyzed using the DNA sequencing software Chromas 2.6.6^4^.

### Immunoblotting

Whole cell extracts were prepared in RIPA buffer [10 mM Tris–Cl (pH 8.0), 1 mM EDTA, 0.1% sodium deoxycholate, 0.1% sodium dodecyl sulfate (SDS), 1% NP40, 140 mM NaCl] supplemented with cOmplete^TM^ Mini Protease Inhibitor Cocktail and PhosSTOP^TM^ EASYpack (both from Roche, Sigma-Aldrich) just prior to use. Protein concentrations of extracts were measured using the BCA Protein Assay Kit (Pierce, Thermo Fisher Scientific) following the standard protocol. 20 μg of protein lysate in loading buffer (62.6 mM Tris–HCl, pH 6.8, 2% SDS, 10% glycerol, 0.01% bromophenol blue) containing 100 mM dithiothreitol (DTT) were boiled for 5 min at 95°C and separated by SDS-PAGE under reducing conditions and blotted on to nitrocellulose membranes (Amersham, Sigma-Aldrich). Following the transfer, membranes were stained in 0.1% amido black solution (MERCK, Schaffhausen, Switzerland) to control for blotting efficiency. After blocking the membranes for 1 h at room temperature in Tris-buffered saline (TBS, pH 7.4) containing 0.05% Tween-20 and 5% skim milk powder (Sigma-Aldrich), they were incubated over-night with primary antibody at 4°C on a rocking platform. Membranes were washed three times in TBS-Tween and incubated for 1 h with peroxidase-conjugated anti-rabbit/mouse IgG in TBS-Tween/5% milk at room temperature. After washing in TBS-Tween, blots were developed using SuperSignal West Dura or Super Signal West Pico PLUS Chemiluminescent Substrate (both from Thermo Fisher Scientific) and scanned by a Chemi Premium Imager Instrument (VWR, Darmstadt, Germany). Primary antibodies used: anti-myc (clone 9E10, Abcam, Cambridge, United Kingdom), anti-IRF6 (clone 14B2C16, BioLegend, San Diego, CA, United States), anti-vinculin (V9131, Sigma-Aldrich), and anti-KLF4 (11880-1-AP, Proteintech).

Some immunoblots were analyzed densitometrically using the ImageJ software. Briefly, the intensity of each protein band was normalized to the vinculin band intensity of the same extract in the same experiment.

### Cloning of the IRF6 Constructs

To generate a myc-tagged IRF6 expression plasmid (IRF6wt), cDNA was synthesized from total RNA extracted from HaCaT cells and PCR was performed on the cDNA using the GoTaq Flexi DNA Polymerase (Promega) with the following primers: 5′-AG*GCGATCGC*CATGGCCCTCCACCCCCGCAGAG-3′/5′- AG*ACGCGT*TTCCGAGTTTACAGTGAAGTGCAG-3′. Pri- mers contained an *Asi*SI (forward) and a *Mlu*I restriction site (reverse), respectively. This allowed the directional cloning into the mammalian expression plasmid pCMV6-A-Puro (Origene, Rockville, MD, United States) containing a myc tag.

For the generation of the specific myc-tagged IRF6_VWS expression plasmid (c.961_965 delGTGTA ins C), the IRF6wt plasmid was used as DNA template for a PCR with the primer pair 5′-AG*GCGATCGC*CATGG CCCTCCACCCCCGCAGAG-3′ (same as above)/5′-GA*CTC GAG*GGTTGGGAGCAACAAGTGATGGGGCACATGGCCCA GACCAGGCTTGCACTGGCACAGCCTGATGGC-3′ (*Xho* I restriction site), allowing directional cloning into the above mentioned pCMV6 plasmid. All constructs were sequence-verified using the sequencing primers CMV (5′-CGCAAATGGGCGGTAGGCGTG-3′) and XL39 (5′-ATTAGG ACAAGGCTGGTGGG-3′).

### Cell Transfections and Treatments

Transient transfections were performed in 35 mm tissue culture dishes with cells at 60–70% confluence using ViaFect (Promega) following the manufacturer’s protocol with 2 μg DNA and 6 μl transfection reagent diluted in OptiMEM (Thermo Fisher Scientific). Six hours after transfection, the transfection mix was removed, and the cells were washed once before being replenished with fresh medium. Cells were analyzed 24 or 48 h post-transfection.

To assess IRF6 stability, 20 μg/ml of the protein translation inhibitor CHX (Sigma-Aldrich) was added to the cells 24 h after transfection. Thereafter, proteins were extracted at the indicated time points and immunoblotted. Proteasome inhibition was induced by 10 μM MG132 (Sigma-Aldrich) for eight hours. When the double treatment MG132/CHX was applied, MG132 was added to the cells 30 min prior to CHX.

### Luciferase Reporter Assay

2.5 × 10^5^ HEK293 cells/ml were seeded into wells of a flat-bottom 96-well plate the day before transfection. On the next morning, cells were transfected as previously described using 90 ng of IFN-Beta_pGL3 [a gift from Nicolas Manel (Addgene plasmid # 102597; http://n2t.net/addgene:102597; RRID:Addgene_102597)], 10 ng of pRL-TK (wild-type *Renilla* luciferase gene for normalization; Promega), and 100 ng of pCMV6-IRF6wt or pCMV6-IRF6_VWS, respectively. Luciferase assays were performed using the Dual-Glo luciferase assays system (Promega) according to their manual. Firefly and Renilla luminescence were read on a Tecan InfinitePro2000 plate reader (Tecan Group Ltd., Männedorf, Switzerland) and the ratio of firefly:renilla luminescence was calculated for each sample and normalized to the reporter-only control.

### Statistical Analysis

Experiments were performed at least three times in multiple replicates. Data were analyzed using GraphPad InStat Software, version 3.05. Data are represented as means ± standard deviation (SD). Multiple comparisons were performed using one-way analysis of variance (ANOVA) with Tukey’s *post hoc* test. Values of *p* ≤ 0.05 were considered significant.

### Data Availability

The raw data supporting the conclusions of this article will be made available by the authors, without undue reservation.

## Results

### VWS Patient-Derived Keratinocytes Display Defects in Terminal Differentiation

**FIGURE 1:**
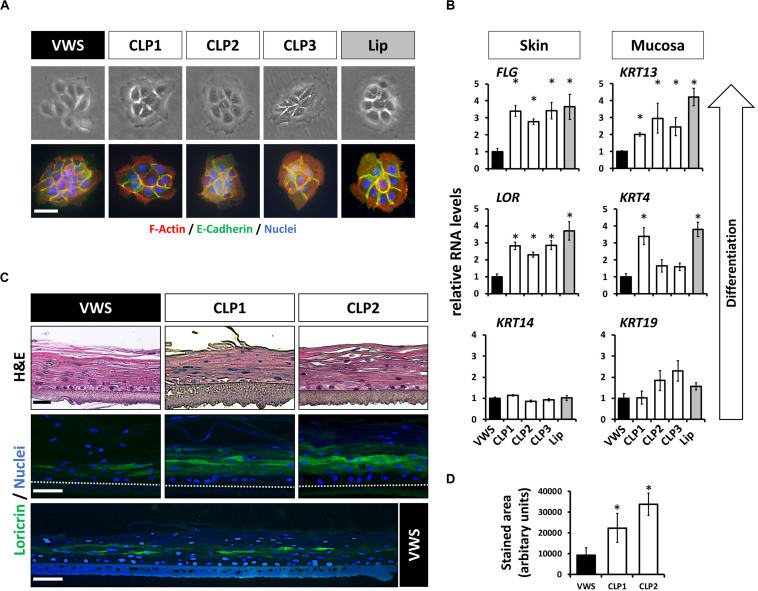
**(A)** Brightfield pictures (top row) and immunofluorescent stainings (bottom row) for E-Cadherin (green) and F-actin (phalloidin, red) of four patient- and one healthy control-derived lip keratinoyte colonies reveal no morphological differences between the VWS patient- and three non-syndromic CLP patient-derived cell cultures (CLP1-3) and the healthy control (lip). Scale bar: 50 μm. **(B)** qPCR analyses of several differentiation markers in VWS-derived keratinocytes (black bar) compared to three non-syndromic CLP-derived keratinocytes (CLP1-3, white bars) and to healthy lip keratinocytes (lip, gray bar). VWS keratinocytes express lower levels of the differentiation markers *LOR* and *FLG* (non-mucosal) as well as of *KRT4* and *KRT13* (mucosal) compared to non-syndromic (CLP1-3) and healthy control lip keratinocytes. In contrast, expression of *KRT14* and *KRT19*, markers of the corresponding basal layers, is similar among the cell cultures tested. Note that our primary lip-derived keratinocytes represent a mixture of both mucosal as well as non-mucosal cells. *p ≤ 0.05 vs. VWS. **(C)** Hematoxylin and eosin (H&E) staining of 3D-skin models using VWS, CLP1, and CLP2 keratinocytes (top row). Scale bar: 50 μm. Staining for LOR indicates differentiation deficiencies of VWS keratinocytes when compared to the others. Scale bar: 50 μm. A larger stretch of the 3D-skin model produced by VWS-keratinocytes is shown at the bottom. Scale bar: 50 μm. Dotted lines represent the upper edge of the inserts. **(D)** Quantification of the LOR-positive area in 3D-skin models reveals a significant difference between VWS and non-syndromic cultures. ^∗^*p* ≤ 0.05 vs. VWS.

Our previous findings suggested that some of the genetic predispositions causing VWS might lead to deficiencies in terminal differentiation of keratinocytes *in vitro* (Degen et al., 2018). To extend these results, we investigated more thoroughly the genetic and phenotypic particularities of VWS-derived lip keratinocytes (VWS) compared to non-syndromic CLP- (CLP1-3) and healthy control-derived lip keratinocytes (lip, Table 1). The characteristic of cell growth, cell size as well as the morphology of the cell colonies were similar in all of the keratinocyte cultures tested as evidenced by brightfield and immunostainings for F-actin (phalloidin) and E-Cadherin (Figure 1A). Next, we wanted to assess their differentiation potential in confluent 2D cultures. As the lip represents a mucosa-skin transition zone, we analyzed differentiation markers of both tissue types, skin and mucosa, by qPCR. We detected lower RNA levels of the differentiation markers *Loricrin* (*LOR*) and *Filaggrin* (*FLG*) (skin) as well as of *Keratin 4* (*KRT4*) and *Keratin 13* (*KRT13*) (mucosa) in VWS keratinocytes compared to the CLP and healthy control lip cells (Figure 1B). In contrast, *Keratin 14* (*KRT14*) and *Keratin 19* (*KRT19*), markers mainly expressed in the basal proliferating layer of keratinized epithelium and oral mucosa, respectively (Lindberg and Rheinwald, 1989), did not significantly differ among the keratinocyte cultures (Figure 1B). We further confirmed a reduced potential to terminally differentiate in VWS keratinocytes using 3D organotypic skin models. While there were no obvious differences visible in H&E stainings of the 3D skin models among the cultures tested, we detected a significantly reduced area of LOR-positive keratinocytes in the suprabasal layers in the VWS culture compared to CLP1 and CLP2 (Figures 1C,D).

### Identification of a Novel IRF6 Variant Causing VWS

**FIGURE 2:**
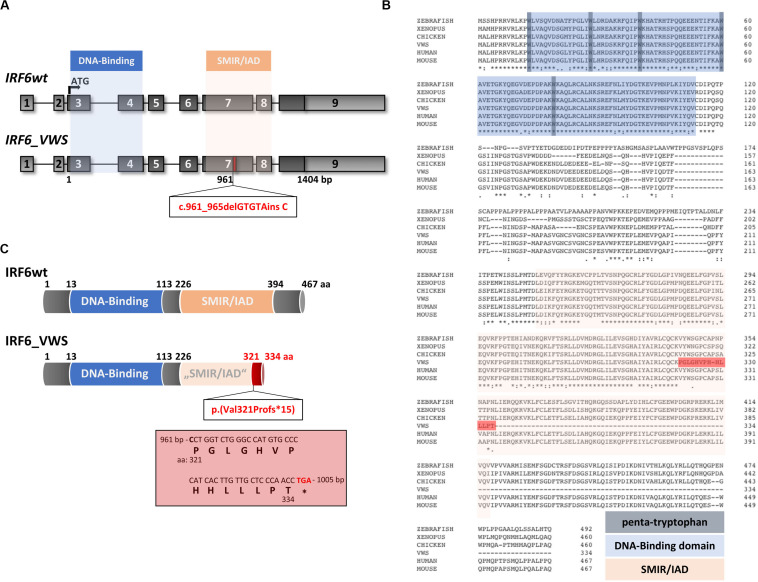
**(A)** Schematic representation of the *IRF6* gene and the novel pathogenic variant *IRF6_VWS* c.961_965delGTGTAinsC (bottom). The *IRF6* gene is located on chromosome 1 (reverse strand), contains nine exons and two crucial domains, the DNA-Binding domain (blue) and the SMAD/IRF (SMIR)/interferon association domain (IAD) (orange). The newly identified gene variant affects exon 7 at base pair (bp) 961, which is indicated by the red box. Light gray boxes: untranslated exons/regions; dark gray boxes: translated exons. **(B)** Sequence alignments of IRF6 in various species. Note that the DNA-Binding domain (blue), including the penta-tryptophan cluster (dark blue), and the SMIR/IAD (light red) are highly conserved. The dark red highlighted stretch indicates the newly generated sequence due to the VWS-causing *IRF6* variant. The numbers indicate the amino acids (aa). **(C)** Schematic representation of the IRF6 and the IRF6_VWS protein p.(Val321Profs*15). Numbers indicate the aa. Note that IRF6_VWS is a truncated *IRF6* variant that contains a unique stretch of 14 aa (box) at its C-terminus. ^∗^stop codon.

Although the VWS keratinocytes displayed deficiencies to terminally differentiate *in vitro* [Figure 1 and (Degen et al., 2018)], the underlying VWS-causing variant in the specific individual remained elusive. Targeted Exome Sequencing (TES) identified the heterozygous frameshift variant c.961_965delGTGTAinsC in *IRF6*. To the best of our knowledge, this *IRF6* variant is novel and has never been described in the literature and is not listed in the Human Gene Mutation Database [HGMD^®^ (Stenson et al., 2017)]. Both parents of the VWS patients are asymptomatic. However, genetical analysis of their *IRF6* genes could not be performed to confirm a *de novo* origin of the variant in the patient. The uncovered variant in our VWS individual affects exon 7 of *IRF6* (Figure 2A), which encodes for the highly conserved SMIR/IAD (Figure 2B). Consequently, the variant results in a predicted amino acid (aa) change at position 321 [p.(Val321Pro)] followed by a frameshift leading to a truncated open reading frame due to the presence of a premature termination codon (PTC) [p.(Val321Profs^∗^15)] in exon 7 (Figures 2A,C). The resulting altered IRF6_VWS protein is predicted to consist of 334 aa only because it lacks the entire C-terminus and the last 73 aa of the SMIR/IAD of the IRF6wt protein. Instead, IRF6_VWS contains a unique C-terminal stretch of 14 aa (Figure 2C, red box). According to the ACMG-standards and guidelines (Richards et al., 2015) this variant is classified as likely pathogenic.

### The IRF6_VWS mRNA Is Targeted by NMD

**FIGURE 3:**
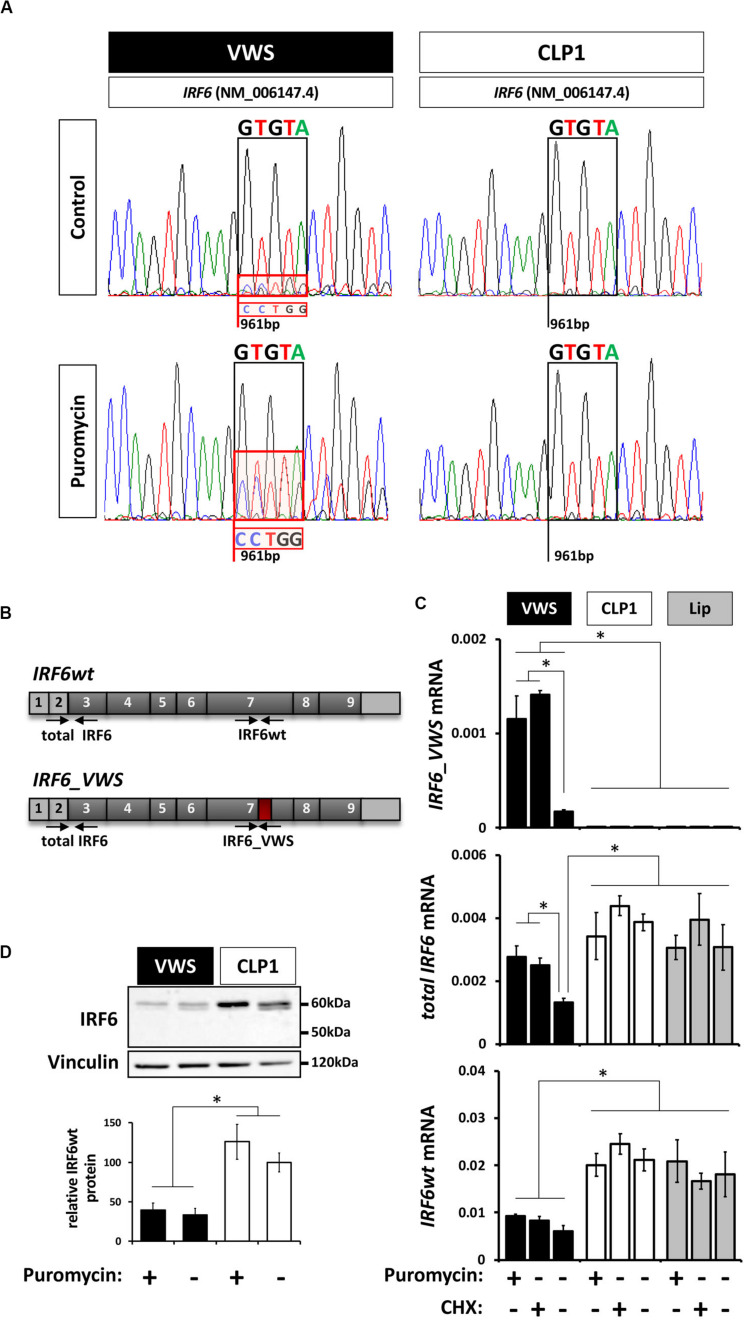
**(A)** Sanger DNA Sequencing chromatograms of the IRF6 sequence encompassing the VWS-causing IRF6 variant (VWS) and the same sequence in a non-syndromic CLP patient (CLP1) in the absence or presence of the non-sense-mediated mRNA decay (NMD) mechanism inhibitor puromycin. Sequences of interest are indicated by boxes. Black box: GTGTA-sequence of *IRF6wt*; red box: CCTGG-sequence of the *IRF6_VWS*. Note that blocking NMD by puromycin results in more pronounced *IRF6_VWS* sequencing peaks in the VWS sample. **(B)** Schematic representation of the *IRF6wt* and *IRF6_VWS* gene with the specific qPCR primers used. Light gray boxes: untranslated exons/regions; dark gray boxes: translated exons; red box: location of mutation and novel sequence. **(C)** qPCR analysis of *IRF6* expression in VWS (black), CLP1 (white), and healthy lip keratinoctyes (gray) in the absence or presence of puromycin and CHX. Note that *IRF6_VWS* is detectable by qPCR and that its levels increase in the presence of both NMD inhibitors, puromycin and CHX (top panel). Also note that *IRF6wt* levels are higher in CLP1 and healthy lip than in VWS (bottom panel). *p ≤ 0.05. **(D)** Immunoblots of protein extracts from VWS (black) and CLP1 (white) keratinocytes in the absence or presence of puromycin for endogenous IRF6. Note that IRF6wt levels are lower in VWS when compared to CLP1 cells and that the endogenous IRF6_VWS is not detectable in the VWS cells. Quantification of IRF6wt levels is shown at the bottom. ^∗^*p* ≤ 0.05. The full-length blots are presented in Supplementary Figure S6.

The non-sense-mediated mRNA decay (NMD) mechanism is known to degrade mRNAs containing PTCs in order to prevent translation of aberrant transcripts (Wilusz et al., 2001; Maquat, 2002). Since our newly identified *IRF6_VWS* allele is transcribed into a transcript with an NMD-triggering PTC [PTC is not located in the last exon and is situated more than ≥ 50–55 bp upstream of the last exon-exon junction (Kurosaki and Maquat, 2016)] (Figure 2), we assessed the possibility of *IRF6_VWS* mRNA being targeted and degraded by NMD. Therefore, we compared the sequencing chromatograms around the *IRF6* variant at position c.961 from fragments that have been amplified from RNA extracted from VWS and CLP1 keratinocytes. A mixed sequencing signal at the position of the novel *IRF6* variant (961 bp) was present in the VWS, but not in the CLP1 keratinocytes (Figure 3A top panel). While the prominent peaks of the chromatogram in VWS correspond to the *IRF6wt* transcript sequence (GTGTA, Figure 3A black box, top panel left), the overlapping smaller peaks additionally disclose the discrete presence of the *IRF6_VWS* transcript sequence (CCTGG, Figure 3A, red box, top panel left). This result would imply that the vast majority (≈ 90%) of the *IRF6_VWS* mRNA is degraded, but that a moderate amount of the VWS-specific transcript remained expressed. To test this speculation and to prove that NMD was the responsible mechanism for the degradation of aberrant *IRF6*, we treated the cells with puromycin, a known NMD inhibitor (Andreutti-Zaugg et al., 1997). Addition of puromycin greatly rescued the diminished levels of *IRF6_VWS* as evidenced by the increased peaks of the mutated IRF6 sequence in the VWS keratinocytes (Figure 3A, red box, bottom panel left). On the other hand, the addition of puromycin did not affect *IRF6* transcripts in CLP1. These results prove that NMD is the responsible mechanism for *IRF6_VWS* degradation.

We further wished to confirm the low expression level of IRF6_VWS and its significant increase by inhibiting NMD in VWS keratinocytes by qPCR and immunoblots. Using primers that showed a higher affinity to anneal to the *IRF6_VWS* than to the *IRF6wt* sequence (Figure 3B), we could reveal that *IRF6_VWS* is expressed and detectable in VWS keratinocytes and that blocking NMD by puromycin and cycloheximide (CHX) significantly increased its levels (Figure 3C, top panel). As expected, the *IRF6_VWS* transcript was not detectable in CLP1- and healthy lip-derived keratinocytes. T*otal IRF6* (*IRF6_VWS* and *IRF6wt*) and *IRF6wt* levels were reduced by 50% in VWS compared to CLP1 and healthy lip keratinocytes. While this reduction could be reversed by puromycin and CHX for *total IRF6*, *IRF6wt* levels did not change in response to the NMD inhibitors (Figure 3C, middle and bottom panels). These results clearly indicate that more *IRF6_VWS* is being expressed in the presence of the NMD inhibitors puromycin and CHX. In agreement with these qPCR findings, we identified significantly higher IRF6wt protein expression in CLP1 compared to VWS keratinocytes, even in the presence of puromycin (Figure 3D). Unfortunately, we are unable to detect the truncated IRF6_VWS protein (≈ 50 kDa), likely due to its very low expression levels.

**FIGURE 4:**
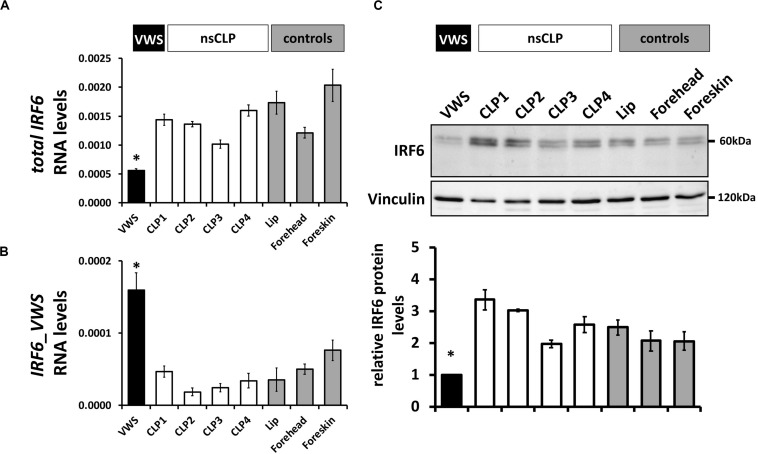
**(A)** qPCR analysis of *IRF6wt* in VWS-keratinocytes (VWS, black), four non-syndromic CLP-keratinocytes (CLP1-4, white) and healthy control keratinocyte cultures (lip, forehead, and foreskin; gray). Note lowest levels of *IRF6wt* in VWS compared to the others. *p ≤ 0.05 vs. CLP1-4, lip, forehead, foreskin. **(B)** qPCR analysis of *IRF6_VWS* in the same keratinocyte cultures. Note that *IRF6_VWS* is only robustly expressed in VWS. *p ≤ 0.05 vs. CLP1-4, lip, forehead, foreskin. **(C)** Immunoblot of the corresponding protein extracts for IRF6 and its densitometric quantification. Note low endogenous IRF6wt protein levels in VWS and that IRF6_VWS, which runs at about 50 kDa, cannot be detected in VWS. ^∗^*p* ≤ 0.05 vs. CLP1-4, lip, forehead, foreskin. The full-length blots are presented in Supplementary Figure S6.

To extend these data, we screened IRF6 mRNA and protein levels in keratinocytes derived from the VWS individual, four non-syndromic CLP patients, and three healthy control tissues (Table 1). While total *IRF6* levels were significantly reduced in VWS compared to all other primary cells tested (Figure 4A), *IRF6_VWS* was only significantly detectable in VWS keratinocytes (Figures 4B), which is consistent with our previous results. Immunoblots confirmed lowest IRF6 protein levels in VWS keratinocytes compared to the other cells tested (Figure 4C), demonstrating that the novel VWS-causing *IRF6* variant results in haploinsufficiency of IRF6 caused by NMD.

### Lower Forced Expression of IRF6_VWS Compared to That of IRF6wt

**FIGURE 5:**
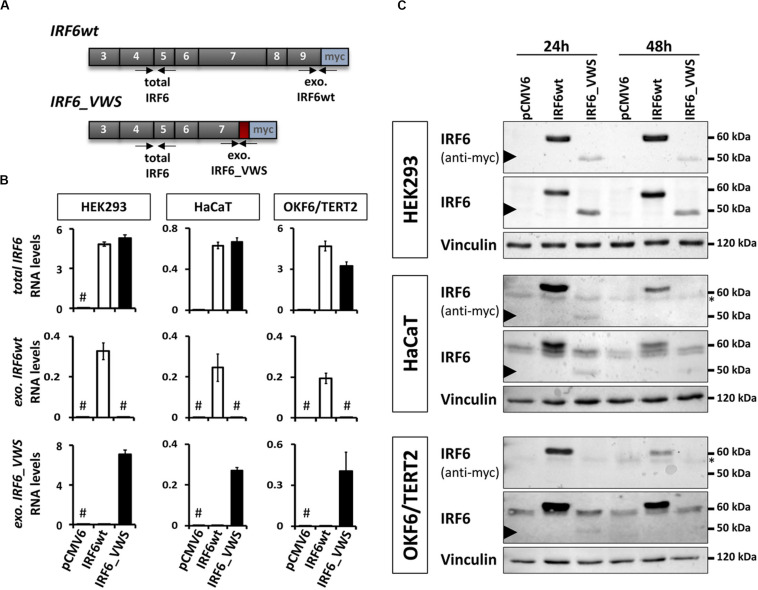
**(A)** Schematic representation of the cloned, exogenous *IRF6wt* and *IRF6_VWS* and the primers used to specifically detect them. Gray boxes: translated exons; red box: *IRF6_VWS* specific mutation; blue box: myc tag; exo.: exogenous. **(B)** qPCR analyses of *total IRF6* (top), *exo. IRF6wt* (middle), and *exo. IRF6_VWS* (bottom) in transiently transfected HEK293, HaCaT and OKF6/TERT2 cells 24 h post-transfection. ^#^not detectable (Ct values > 32). **(C)** Immunoblots of transiently transfected HEK293 (top), HaCaT (middle), and OKF6/TERT2 (bottom) 24 and 48 h after transfection with an anti-IRF6 and an anti-myc antibody. Note that while IRF6wt runs at 60 kDa, IRF6_VWS is weakly expressed at 50 kDa (arrowhead). ^∗^background band. The full-length blots are presented in Supplementary Figure S6.

Since the NMD-dependent degradation of *IRF6_VWS* is not 100% efficient, a small amount of the aberrant transcript remains expressed and detectable (Figures 3, 4). This prompted us to investigate the molecular characteristics of the ectopically expressed IRF6_VWS protein in HEK293, HaCaT and OKF6/TERT2 cell lines. Using specific qPCR primers recognizing *exogenous IRF6wt* and *exogenous IRF6_VWS* (Figure 5A), we could readily notice robust expression of both *IRF6wt* and *IRF6_VWS* in all three cell lines 24 h after transfection (Figure 5B). In addition, while *IRF6* was endogenously expressed in HaCaT and OKF6/TERT2 cells, *IRF6* could not be detected in HEK293 cells (Figure 5B). Parallel cultures were analyzed by immunoblots for IRF6 protein levels 24 and 48 h post-transfection. IRF6wt was strongly over-expressed in all three cell lines after 24 h and it was still prominently detectable after 48 h (Figure 5C). In contrast, IRF6_VWS was only poorly expressed 24 h post-transfection and its forced expression was completely lost after 48 h in HaCaT and OKF6/TERT2 cells (Figure 5C, arrowheads and Supplementary Figure S1).

### IRF6_VWS Is Mislocalized in Cells

**FIGURE 6:**
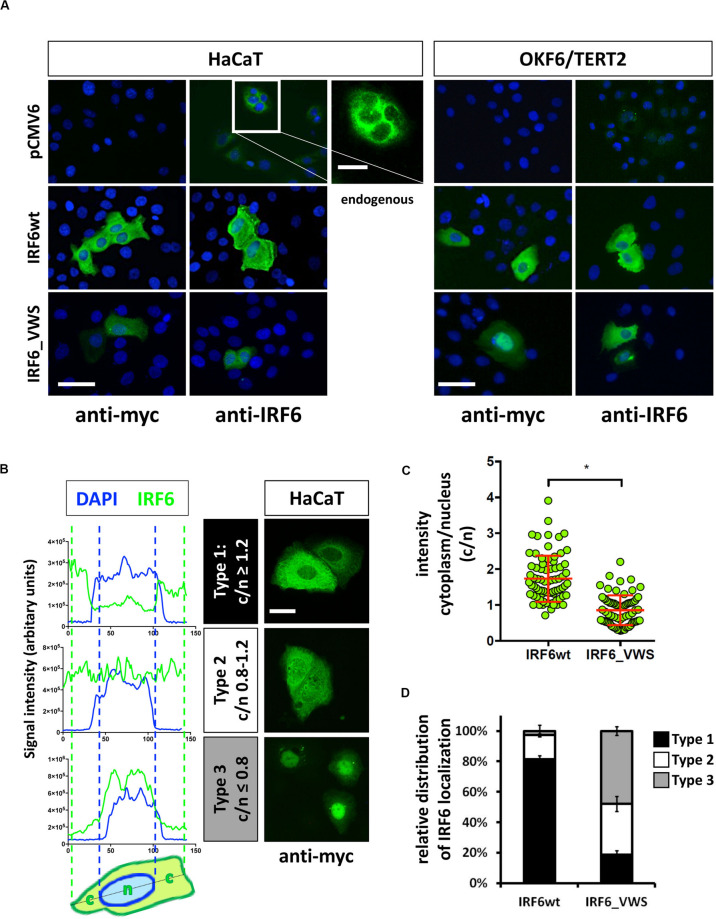
**(A)** HaCaT and OKF6/TERT2 cells were transiently transfected with pCMV6, IRF6wt, and IRF6_VWS and stained with an anti-myc and anti-IRF6 antibody to analyze the localization of the exogenous proteins. Scale bar: 20 μm. Endogenous IRF6 was weakly detectable in both cell lines (pCMV6/anti-IRF6 panels) and is shown for HaCaT cells as a close-up (longer exposure). Scale bar: 10 μm. Note that IRF6_VWS was weaker expressed than IRF6wt. **(B)** Representative linescan plots (blue: DAPI/nucleus channel; green: FITC/IRF6 channel) of individual cells (left) describing the following three types of IRF6 localization: Type 1: c/n ≥ 1.2; Type 2: c/n = 0.8–1.2; Type 3: c/n ≤ 0.8 (middle). C: cytoplasmic intensity; n: nuclear intensity. Matching IRF6 localizations for each of the three categories are shown as pictures of anti-myc immunostained IRF6-transfected HaCaT cells (right). Scale bar: 10 μm. **(C)** Dot plots summarizing the analysis of 75 individual IRF6wt- and IRF6_VWS-transfected HaCaT cells by linescan plots showing the c/n ratio. Note that in IRF6_VWS-transfected cells there is a prominent fraction of cells with a ratio c/n ≤ 0.8 (Type 3). ^∗^*p* ≤ 0.001. **(D)** Relative distribution of exogenous IRF6 localization within transfected HaCaT cells. Note that while most (≈ 80%) of the IRF6wt is localized in the cytoplasm (Type 1: c/n ≥ 1.2), most (≈ 50%) of the IRF6_VWS is found in the nucleus (Type 3: c/n ≤ 0.8).

Next we wanted to analyze the cellular localization of IRF6_VWS in keratinocytes and whether its localization differs to the one of IRF6wt. Exogenous IRF6wt was mostly found expressed in the cytoplasm of both HaCaT and OKF6/TERT2 cells, which is in agreement with its endogenous localization (Figure 6A). The localization of IRF6_VWS was slightly different than its wt counterpart as its weaker expression was detected more often in the nucleus (Figure 6A and Supplementary Figure S2). To clarify this initial observation, we analyzed 75 individual IRF6wt and IRF6_VWS-transfected HaCaT cells for the localization of exogenous IRF6. We used linescan plots and determined the exogenous IRF6 signal intensity in the cytoplasm (c) and in the nucleus (n) for each of the transfected cells. This allowed us to calculate the c/n ratio for each individual cell, based upon which we could define three different IRF6 localization patterns: Type 1, c/n ≥ 1.2 (mostly cytoplasmic); Type 2, c/n = 0.8–1.2 (within all the cell); Type 3, c/n ≤ 0.8 (mostly nuclear). Representative linescan plots of the three IRF6 localization types and the corresponding immunostainings of IRF6_VWS-transfected cells are shown in Figure 6B. Our analysis revealed a significant lower mean c/n ratio in cells transfected with IRF6_VWS than with IRF6wt (Figure 6C), suggesting enhanced nuclear presence of IRF6_VWS, which is in agreement with our initial visual observations. While 80% of the IRF6wt-transfected cells exclusively expressed IRF6wt in the cytoplasm (Type 1), the majority (80%) of ectopic IRF6_VWS was found in the nucleus (Type 2 and 3) of transfected cells (Figure 6D).

### IRF6_VWS Is Less Stable Than IRF6wt

**FIGURE 7:**
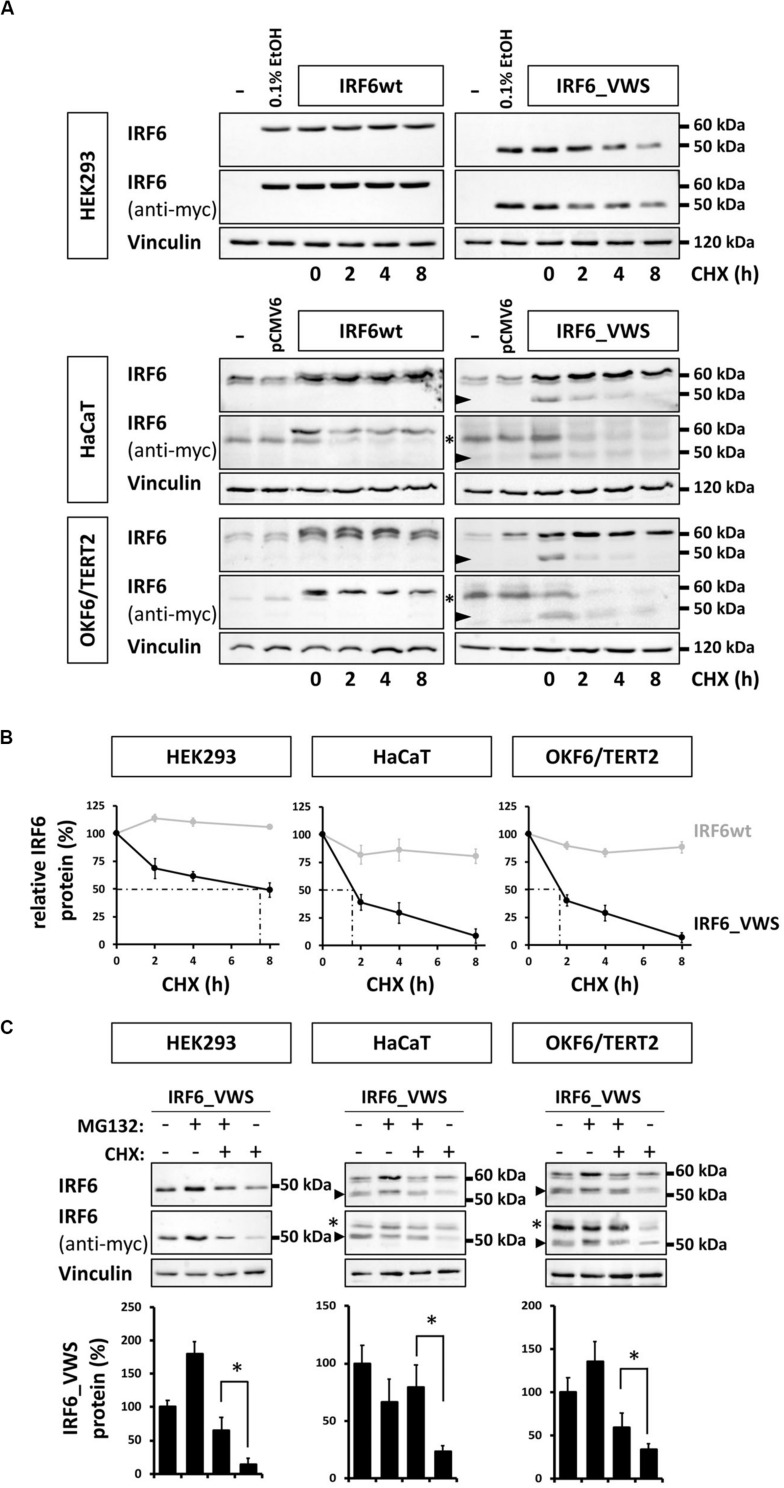
**(A)** Immunoblots of IRF6 in IRF6wt- and IRF6_VWS-transfected HEK293, HaCaT, and OKF6/TERT2 cells treated with the protein synthesis inhibitor cycloheximide (CHX). While IRF6wt was stable within 8 h of CHX treatment, IRF6_VWS levels were greatly reduced. ^∗^background band. Arrowheads: IRF6_VWS. **(B)** Quantification of the immunoblots (anti-myc antibody) confirms that IRF6_VWS (black) is less stable than IRF6wt (gray). **(C)** Immunoblots of IRF6_VWS-transfected cells in the presence of the proteasome inhibitor MG132, CHX, and combination of MG132/CHX for levels of IRF6_VWS. ^∗^background band. The quantifications of the immunoblots are shown below. Note that presence of MG132 greatly diminishes the effect of CHX on IRF6_VWS. ^∗^*p* ≤ 0.05. Arrowheads: IRF6_VWS. The full-length blots are presented in Supplementary Figure S6.

Our observations of consistently weaker expression of exogenous IRF6_VWS compared to IRF6wt (Figures 5C, 6A) prompted us to test the hypothesis that IRF6_VWS is less stable than IRF6wt. Therefore, we used a CHX chase assay to follow the levels (≈ stability) of the two proteins. As shown in Figure 7A (left panels), immunoblots of cell lysates revealed that exogenous IRF6wt levels remained stable within eight hours of CHX treatment in all cell lines tested. In contrast, IRF6_VWS levels were already significantly reduced in the presence of CHX after two hours and almost absent after eight hours (Figure 7A, arrowheads, right panels). Quantification of the blots disclosed a half-life for IRF6_VWS of two hours in the two keratinocyte cell lines, and of around seven hours in HEK293 cells (no endogenous IRF6) (Figure 7B and Supplementary Figure S3).

Next, we tested whether inhibiting the proteasome system could prevent the rapid degradation of IRF6_VWS observed after CHX treatment. IRF6_VWS transfected cells were either treated with the proteasome inhibitor MG132 and CHX alone or in combination for eight hours. Immunoblot analysis demonstrated that MG132 was able to significantly inhibit CHX-mediated degradation of IRF6_VWS in all cell lines tested (Figure 7C, arrowheads), suggesting that IRF6_VWS is degraded in a proteasome-dependent manner. Consistently, almost all cell lines showed a moderate increase of IRF6_VWS in response to MG132 (Figure 7C and Supplementary Figure S4).

### IRF6_VWS Maintains Transcription Factor Function

**FIGURE 8:**
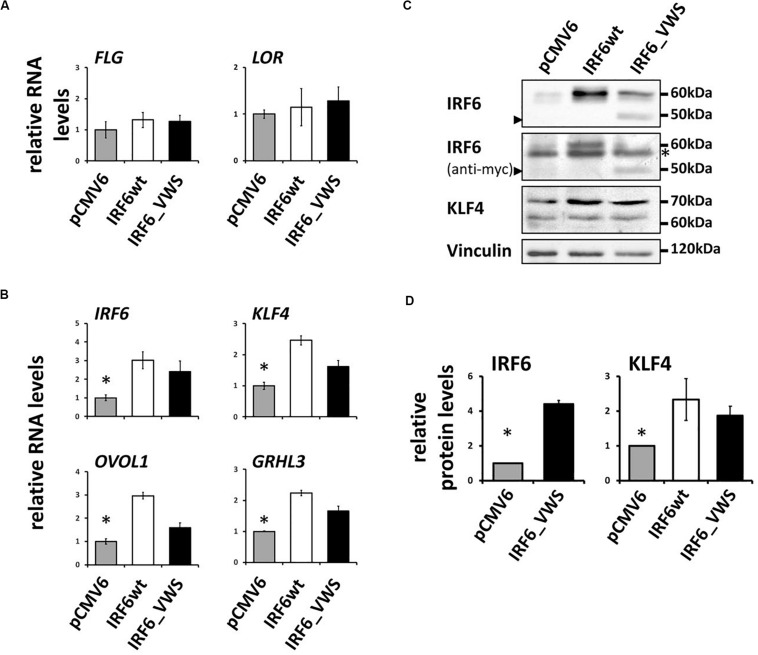
**(A)** qPCR analysis for endogenous *LOR* and *FLG* in HaCaT cells transfected with IRF6wt and IRF6_VWS 24 h after transfection. Note that neither *LOR* nor *FLG* are induced by forced expression of IRF6. **(B)** qPCR analysis of endogenous *IRF6*, *GRHL3*, *KLF4*, and *OVOL1* in HaCaT cells in response to IRF6wt and IRF6_VWS over-expression. *p ≤ 0.05 vs. IRF6wt, IRF6_VWS. Note that the forward primer for the detection of endogenous IRF6 using qPCR was designed to amplify the non-coding 5’UTR region of the *IRF6* gene. **(C)** Immunoblots for endogenous IRF6 and KLF4 in HaCaT cells transfected with IRF6wt and IRF6_VWS. Note that IRF6_VWS is able to induce these proteins, although a little less effective than IRF6wt. ^∗^background band. Arrowheads: IRF6_VWS. The full-length blots are presented in Supplementary Figure S6. **(D)** Quantification of the immunoblots. ^∗^*p* ≤ 0.05 vs. IRF6wt, IRF6_VWS.

Finally, we aimed to study whether IRF6_VWS, although lacking part of its SMIR/IAD, can still act as a transcription factor and regulate gene expression. Initially, we used a luciferase assay approach and could show that both IRF6wt and IRF6_VWS were able to activate an Interferon-β promoter construct (Supplementary Figure S5). However, at the end we were keen to learn whether IRF6_VWS was able to modulate endogenous target gene expression. Although, IRF6 is known to regulate epidermal differentiation, forced expression of neither IRF6wt nor IRF6_VWS was sufficient to induce the late differentiation markers *LOR* and *FLG* (Figure 8A), which is in agreement with a study performed in mice (Biggs et al., 2012). However, we found significant induction of endogenous *IRF6* itself, *GRHL3*, *KLF4*, and *OVOL1* in response to both ectopic IRF6wt and IRF6_VWS expression (Figure 8B). All of these transcription factor genes have been reported to be direct IRF6 targets (Botti et al., 2011; de La Garza et al., 2013; Liu et al., 2016), which is in line with our own data (Figure 8B). We validated some of these results on protein levels by immunoblotting for IRF6 and KLF4. Forced expression of IRF6_VWS, as evidenced by the presence of the truncated IRF6_VWS protein at 50 kDa (arrowhead), as well as ectopic IRF6wt expression resulted in increased levels of endogenous IRF6 at 60 kDa and of KLF4 (Figures 8C,D). Generally, the mRNA and protein induction in response to forced IRF6_VWS expression was slightly lower than the one to IRF6wt (Figures 8B,D). Still, our data demonstrate that IRF6_VWS maintains transcription factor function.

## Discussion

The basis for this study was our previous work that showed variations in the potential to terminally differentiate among CLP-derived keratinocytes. One patient whose keratinocytes exhibited defects in the epidermal differentiation program presented with lower lip pits and CLP, strong clinical diagnostic criteria for VWS (Rizos and Spyropoulos, 2004; Degen et al., 2018). With an estimated frequency of about one in 35’000 newborns, VWS is a very rare syndrome (Dixon et al., 2011). Hence, the availability and the possibility to work with VWS patient-derived keratinocytes, which are isolated from the site of the orofacial defect collected during primary lip repair, is infrequent and makes our study very unique and relevant. Using a combinatorial approach of genetics as well as molecular and cellular tools, we present data on the mechanism by which a novel pathogenic *IRF6* variant causes VWS.

The VWS locus was initially mapped to chromosome 1q32-41 (Murray et al., 1990; Schutte et al., 2000) and the gene encoding for the transcription factor IRF6 was later found to be mutated in most of the VWS cases (Kondo et al., 2002). Genetic analysis of our VWS individual revealed a novel VWS-causing *IRF6* variant c.961_965delGTGTAinsC/p.(Val321Profs^∗^15). According to HGMD^®^ (Stenson et al., 2017), there are currently 351 described pathogenic *IRF6* variants that either cause non-syndromic CLP, or the rare diseases Pierre Robin Sequence (OMIM # 261800), VWS or the related PPS. These pathogenic variants are non-randomly distributed among the nine exons of *IRF6* as most of them are localized within its two conserved functional domains, the DBD (aa 13-113, exons 3–4) and the SMIR/IAD (aa 226-394, exons 7–8). Whereas the distribution of the *IRF6* variants associated with VWS are balanced between the two important domains of IRF6, most of the missense variants causing PPS are present within the DBD (Kondo et al., 2002). Our newly identified *IRF6* pathogenic variant is located in the SMIR/IAD (Figure 2) and results in a truncated version of IRF6, lacking its C-terminus, which contains two phosphorylation sites at Ser413 and Ser424 important for IRF6 activation (Chen et al., 2008; Tamura et al., 2008; Oberbeck et al., 2019). Based on the observations that IRF6 truncations are more common in VWS than in PPS (Kondo et al., 2002; de Lima et al., 2009) and that deletions within the VWS locus have already been described a long time ago in VWS-affected families (Bocian and Walker, 1987; Schutte et al., 1999; Osoegawa et al., 2008), haploinsufficiency of IRF6 is assumed to be the reason for IRF6-caused VWS. Using our patient-derived keratinocytes, we could prove that our newly discovered pathogenic *IRF6* variant indeed results in haploinsufficiency of IRF6 in the VWS keratinocytes (Figure 4). *IRF6_VWS* mRNA undergoes an NMD process, which markedly degrades the aberrant transcript (Figure 3). NMD is a cellular surveillance pathway that reduces gene expression by eliminating mRNA transcripts containing premature PTCs (Schweingruber et al., 2013). The ability to discriminate PTC-containing mRNA in mammalians from mRNAs with normal termination codons is dependent on the position of the PTC within the mRNA. Mutant transcripts with a PTC in the last exon, or within 50–55 bp upstream of the last exon-exon junction are not recognized as NMD substrates and truncated mutant proteins are expected to be translated from these aberrant mRNAs. In contrast, a PTC that is not located in the last exon, or more than 50–55 bp upstream of the last exon-exon junction is recognized and degraded by the NMD process (Nagy and Maquat, 1998; Byers, 2002; Kurosaki and Maquat, 2016; Popp and Maquat, 2016). Our identified pathogenic *IRF6* variant results in a PTC that is situated in the third last exon (exon 7) and more than 50–55 bp upstream from the last exon-exon junction. Hence, the PTC in our novel IRF6 variant adheres to the rules of NMD. Notably, NMD can be beneficial for us by eliminating the synthesis of C-terminally truncated proteins with dominant-negative effects. However, if the truncated protein encoded by the PTC-containing mRNA remains some residual physiological function, NMD-mediated degradation of mRNA abundance might cause more clinical problems than benefits (Schweingruber et al., 2013). These circumstances reflect the situation in our VWS case: (1) the aberrant *IRF6* transcript contains an NMD-triggering PTC in the third last exon (exon 7) of *IRF6* (Figure 2) and (2) the IRF6_VWS protein still harbors some physiological transcription factor activity as it is able to induce the endogenous transcription factors *GRHL3*, *KLF4*, and *OVOL1* as well as *IRF6* itself (Figure 8). There is increasing evidence that a IRF6 threshold level is required for its full physiological activity during craniofacial development (Ingraham et al., 2006; Richardson et al., 2006), neurulation (Kousa et al., 2019), and keratinocyte migration and wound healing (Rhea et al., 2020). Therefore, the efficiency of the NMD process might be a crucial modulator of the clinical manifestation of VWS, a condition often associated with truncating *IRF6* variants (de Lima et al., 2009). A less effective NMD in our VWS individual would result in an increased amount of aberrant *IRF6_VWS* mRNA and consequently, to higher levels of IRF6_VWS protein, which maintains some transcription factor function (Figure 8). This increase in IRF6 protein levels might be enough to reach the IRF6 threshold required for its proper functioning. However, since VWS represents a developmental malformation and NMD is an important regulator of many physiological mRNAs, therapeutic treatments to suppress translation termination at the PTC, allowing the generation of a truncated IRF6_VWS protein, are not feasible (Hwang and Maquat, 2011; Huang and Wilkinson, 2012; Karam et al., 2013; Kurosaki and Maquat, 2016).

IRF6 regulates the balance between keratinocyte proliferation/differentiation. *Irf6*-deficient mice exhibit a hyperproliferative epidermis that fails to undergo terminal differentiation leading to defects during craniofacial development (Kondo et al., 2002; Ingraham et al., 2006; Richardson et al., 2006). These IRF6-related phenotypes have been recently confirmed in humans using skin biopsies as well as keratinocytes isolated from the hip region of VWS patients harboring *IRF6* variants (Hixon et al., 2016). These observations are in agreement with our own results using primary VWS lip keratinocytes, which exhibit a higher proliferation rate (Degen et al., 2018) and defects in terminal differentiation (Figure 1). For the first time, we used such primary CLP patient-derived keratinocytes in 3D-skin models. We could show that both the VWS- and non-syndromic CLP-derived keratinocytes were able to differentiate, with no obvious morphological differences, as evidenced by H&E staining. This apparent discrepancy to the *in vivo* situation in VWS skin tissues [e.g., thicker epidermis in VWS patients compared to non-syndromic CLP patient (Hixon et al., 2016)] might reflect the technical limits of *in vitro* 3D-cultures, which still represent very simplified skin models. Yet, we uncovered subtle defects in the late epidermal differentiation program in VWS-derived keratinocytes compared to CLP keratinocytes, such as reduced levels of LOR in 3D-skin models (Figure 1).

The pathogenic *IRF6* variant (c.961_965delGTGTAinsC) results in IRF6_VWS being expressed at significantly reduced levels compared to IRF6wt due to reduced protein stability (Figure 7). Similar results have been reported for another VWS-causing IRF6 variant, p.(Arg412X) (Kwa et al., 2015). This observation might be explained by studies on the structure of IRF3 showing that the IAD forms a condensed hydrophobic core with its flanking autoinhibitory elements, which is then bound by the DBD resulting in a compact structure of IRF3 (Qin et al., 2003). IRF6 is known to be similarly inhibited by autoinhibitory elements located in its C-terminal domain (Little et al., 2009). Hence, lack of the C-terminus of IRF6, as is the case in our VWS patient, might lead to a relatively open and extended structure of IRF6 that is prone to degradation (Kwa et al., 2015). We further show that degradation of IRF6_VWS is proteasome-dependent, an observation that is also supported by other studies (Bailey et al., 2008; Kwa et al., 2015).

Endogenous IRF6 as well as exogenous IRF6wt are mostly localized in the cytoplasm of cells. This is in contrast to IRF6_VWS, which was often detected in the nucleus (Figure 6). However, the biological consequences of this apparent mislocalization remain to be determined. Latent transcription factors often reside in the cytoplasm and translocate to the nucleus upon specific external stimuli. This shuttling requires nuclear localization signals (NLS), nuclear export signals (NES), and binding of chaperones. To our knowledge, such signals and chaperones have not been identified and verified for IRF6 so far. We can only speculate about the reasons for our observation of enhanced nuclear presence of IRF6_VWS: (1) there is a NES present in the C-terminal region of IRF6 that is missing in our VWS patient due to the gene variant; (2) nuclear export of IRF6 might depend on certain proteins (chaperones) that interact with and guide IRF6 out of the nucleus. The truncating IRF6 variant might abolish these binding sites, which results in more nuclear IRF6_VWS; (3) the newly generated very C-terminal aa sequence of IRF6_VWS, as a result of the mutation, mimics a new NLS, which shifts the steady state equilibrium to the nucleus; (4) posttranslational modifications (e.g., phosphorylations, glycosylations) within the missing sequence of IRF6_VWS are required for nuclear export. Clearly, our initial observations warrant further studies to exactly elucidate the shuttling of IRF6 in and out of the nucleus.

In summary, we identified a novel pathogenic *IRF6* variant c.961_965delGTGTAinsC/p.(Val321Profs^∗^15) in a VWS individual and show how this *IRF6* variant is regulated by an NMD process and affects the phenotype of keratinocytes: most of the *IRF6_VWS* mRNA is subject to NMD, resulting in reduced IRF6 levels (haploinsufficiency). The minor amount of *IRF6_VWS* that is not degraded by NMD is translated into a functional transcription factor that can still regulate GRNs important for epidermal differentiation such as *GRHL3*, *KLF4*, *OVOL1*, and *IRF6* (Figure 8). However, a greatly accelerated IRF6_VWS turnover as well as protein mislocalization further complicate proper IRF6 function. At the end, IRF6 is not present at the required threshold level to be able to properly fulfill all its important activities. Such defects might be reflected in phenotypic changes in cells during development, such as reduced formation of the periderm, whose structure is dependent on IRF6 function (Richardson et al., 2009, 2014). An improperly arranged and harmed periderm cannot prevent pathological epithelial fusions between different tissues in the oral cavity anymore (Hammond et al., 2019), which might explain the clinical clefting manifestation in our VWS individual. Finally, our study not only contributes to the advancement of knowledge on IRF6 function in health and disease, but also promotes the following idea: although the use of postnatal tissue to study CLP that arises early in development comes with some challenges and assumptions, various cellular and molecular properties persist postnatally in CLP patient-derived cells, which makes studies using such primary cells very unique and clinically relevant.

## Data Availability Statement

Datasets are available on request. The raw data supporting the conclusions of this article will be made available by the authors, without undue reservation.

## Ethics Statement

The studies involving human participants were reviewed and approved by Kantonale Ethikkommission für die Forschung, Bern. Written informed consent to participate in this study was provided by the participants’ legal guardian/next of kin.

## Author Contributions

EG, JF, LP, and MD performed the experiments and analyzed the data. AS was responsible for the genetic analyses and their interpretation. MD planned, coordinated and designed the experiments and wrote the manuscript. CK critically revised the manuscript and provided support throughout the project. All clinical work was performed by GL and IS. All authors critically reviewed the manuscript.

## Conflict of Interest

The authors declare that the research was conducted in the absence of any commercial or financial relationships that could be construed as a potential conflict of interest.

## Footnotes

1 https://imagej.nih.gov/ij/download.html

2 www.pga.mgh.harvard.edu

3 www.ncbi.nlm.nih.gov/tools/primer-blast

4 www.technelysium.com.au

## References

[B1] Andreutti-Zaugg C. Scott R. J. Iggo R. (1997). Inhibition of nonsense-mediated messenger Rna decay in clinical samples facilitates detection of human Msh2 mutations with an in vivo fusion protein assay and conventional techniques. *Cancer Res.* 57 3288–3293. 9242462

[B2] Bailey C. M. Abbott D. E. Margaryan N. V. Khalkhali-Ellis Z. Hendrix M. J. (2008). Interferon regulatory factor 6 promotes cell cycle arrest and is regulated by the proteasome in a cell cycle-dependent manner. *Mol. Cell Biol.* 28 2235–2243. 10.1128/MCB.01866-07 18212048 PMC2268429

[B3] Biggs L. C. Rhea L. Schutte B. C. Dunnwald M. (2012). Interferon regulatory factor 6 is necessary but not sufficient for keratinocyte differentiation. *J. Invest. Dermatol.* 132 50–58. 10.1038/jid.2011.272 21918538 PMC3237898

[B4] Bocian M. Walker A. P. (1987). Lip pits and deletion 1q32—-41. *Am. J. Med. Genet.* 26 437–443. 10.1002/ajmg.1320260223 3812594

[B5] Botti E. Spallone G. Moretti F. Marinari B. Pinetti V. Galanti S. (2011). Developmental factor IRF6 exhibits tumor suppressor activity in squamous cell carcinomas. *Proc. Natl. Acad. Sci. U.S.A.* 108 13710–13715. 10.1073/pnas.1110931108 21807998 PMC3158164

[B6] Bush J. O. Jiang R. (2012). Palatogenesis: morphogenetic and molecular mechanisms of secondary palate development. *Development* 139 231–243. 10.1242/dev.067082 22186724 PMC3243091

[B7] Byers P. H. (2002). Killing the messenger: new insights into nonsense-mediated mRNA decay. *J. Clin. Invest.* 109 3–6. 10.1172/JCI0214841 11781342 PMC150830

[B8] Chen W. Lam S. S. Srinath H. Jiang Z. Correia J. J. Schiffer C. A. (2008). Insights into interferon regulatory factor activation from the crystal structure of dimeric IRF5. *Nat. Struct. Mol. Biol.* 15 1213–1220. 10.1038/nsmb.1496 18836453 PMC2757928

[B9] de La Garza G. Schleiffarth J. R. Dunnwald M. Mankad A. Weirather J. L. Bonde G. (2013). Interferon regulatory factor 6 promotes differentiation of the periderm by activating expression of Grainyhead-like 3. *J. Invest. Dermatol.* 133 68–77. 10.1038/jid.2012.269 22931925 PMC3541433

[B10] de Lima R. L. Hoper S. A. Ghassibe M. Cooper M. E. Rorick N. K. Kondo S. (2009). Prevalence and nonrandom distribution of exonic mutations in interferon regulatory factor 6 in 307 families with Van der Woude syndrome and 37 families with popliteal pterygium syndrome. *Genet. Med.* 11 241–247. 10.1097/GIM.0b013e318197a49a 19282774 PMC2789395

[B11] Degen M. Natarajan E. Barron P. Widlund H. R. Rheinwald J. G. (2012). MAPK/ERK-dependent translation factor hyperactivation and dysregulated laminin gamma2 expression in oral dysplasia and squamous cell carcinoma. *Am. J. Pathol.* 180 2462–2478. 10.1016/j.ajpath.2012.02.028 22546478 PMC3378915

[B12] Degen M. Wiederkehr A. La Scala G. C. Carmann C. Schnyder I. Katsaros C. (2018). Keratinocytes isolated from individual cleft lip/palate patients display variations in their differentiation potential in vitro. *Front. Physiol.* 9:1703. 10.3389/fphys.2018.01703 30555344 PMC6281767

[B13] Deshpande A. S. Goudy S. L. (2019). Cellular and molecular mechanisms of cleft palate development. *Laryngoscope Investig. Otolaryngol.* 4 160–164. 10.1002/lio2.214 30828634 PMC6383315

[B14] Dickson M. A. Hahn W. C. Ino Y. Ronfard V. Wu J. Y. Weinberg R. A. (2000). Human keratinocytes that express hTERT and also bypass a p16(INK4a)-enforced mechanism that limits life span become immortal yet retain normal growth and differentiation characteristics. *Mol. Cell Biol.* 20 1436–1447. 10.1128/MCB.20.4.1436-1447.2000 10648628 PMC85304

[B15] Dixon M. J. Marazita M. L. Beaty T. H. Murray J. C. (2011). Cleft lip and palate: understanding genetic and environmental influences. *Nat. Rev. Genet.* 12 167–178. 10.1038/nrg2933 21331089 PMC3086810

[B16] Eroshkin A. Mushegian A. (1999). Conserved transactivation domain shared by interferon regulatory factors and Smad morphogens. *J. Mol. Med.* 77 403–405. 10.1007/s001090050369 10426188

[B17] Gritli-Linde A. (2007). Molecular control of secondary palate development. *Dev. Biol.* 301 309–326. 10.1016/j.ydbio.2006.07.042 16942766

[B18] Hammond N. L. Dixon J. Dixon M. J. (2019). Periderm: Life-cycle and function during orofacial and epidermal development. *Semin. Cell Dev. Biol.* 91 75–83. 10.1016/j.semcdb.2017.08.021 28803895

[B19] Hixon K. Rhea L. Standley J. Canady F. J. Canady J. W. Dunnwald M. (2016). Interferon regulatory factor 6 controls proliferation of keratinocytes from children with Van der Woude syndrome. *Cleft Palate Craniofac. J.* 54 281–286. 10.1597/15-275 27115562 PMC5776006

[B20] Hortis-Dzierzbicka M. Radkowska E. Fudalej P. S. (2012). Speech outcomes in 10-year-old children with complete unilateral cleft lip and palate after one-stage lip and palate repair in the first year of life. *J. Plast. Reconstr. Aesthet. Surg.* 65 175–181. 10.1016/j.bjps.2011.09.015 21978731

[B21] Huang L. Wilkinson M. F. (2012). Regulation of nonsense-mediated mRNA decay. *Wiley Interdiscip. Rev. RNA* 3 807–828. 10.1002/wrna.1137 23027648

[B22] Hwang J. Maquat L. E. (2011). Nonsense-mediated mRNA decay (n.d.) in animal embryogenesis: to die or not to die, that is the question. *Curr. Opin. Genet. Dev.* 21 422–430. 10.1016/j.gde.2011.03.008 21550797 PMC3150509

[B23] Ingraham C. R. Kinoshita A. Kondo S. Yang B. Sajan S. Trout K. J. (2006). Abnormal skin, limb and craniofacial morphogenesis in mice deficient for interferon regulatory factor 6 (Irf6). *Nat. Genet.* 38 1335–1340. 10.1038/ng1903 17041601 PMC2082114

[B24] Jones J. L. Canady J. W. Brookes J. T. Wehby G. L. L’heureux J. Schutte B. C. (2010). Wound complications after cleft repair in children with Van der Woude syndrome. *J. Craniofac. Surg.* 21 1350–1353. 10.1097/SCS.0b013e3181ec6aad 20856020 PMC3018692

[B25] Juriloff D. M. Harris M. J. (2008). Mouse genetic models of cleft lip with or without cleft palate. *Birth Defects Res. A Clin. Mol. Teratol.* 82 63–77. 10.1002/bdra.20430 18181213

[B26] Karam R. Wengrod J. Gardner L. B. Wilkinson M. F. (2013). Regulation of nonsense-mediated mRNA decay: implications for physiology and disease. *Biochim. Biophys. Acta* 1829 624–633. 10.1016/j.bbagrm.2013.03.002 23500037 PMC3660545

[B27] Kondo S. Schutte B. C. Richardson R. J. Bjork B. C. Knight A. S. Watanabe Y. (2002). Mutations in IRF6 cause Van der Woude and popliteal pterygium syndromes. *Nat. Genet.* 32 285–289. 10.1038/ng985 12219090 PMC3169431

[B28] Kousa Y. A. Schutte B. C. (2016). Toward an orofacial gene regulatory network. *Dev. Dyn.* 245 220–232. 10.1002/dvdy.24341 26332872 PMC4755791

[B29] Kousa Y. A. Zhu H. Fakhouri W. D. Lei Y. Kinoshita A. Roushangar R. R. (2019). The TFAP2A-IRF6-GRHL3 genetic pathway is conserved in neurulation. *Hum. Mol. Genet.* 28 1726–1737. 10.1093/hmg/ddz010 30689861 PMC6494790

[B30] Kurosaki T. Maquat L. E. (2016). Nonsense-mediated mRNA decay in humans at a glance. *J. Cell Sci.* 129 461–467. 10.1242/jcs.181008 26787741 PMC4760306

[B31] Kwa M. Q. Huynh J. Reynolds E. C. Hamilton J. A. Scholz G. M. (2015). Disease-associated mutations in IRF6 and RIPK4 dysregulate their signalling functions. *Cell Signal.* 27 1509–1516. 10.1016/j.cellsig.2015.03.005 25784454

[B32] Lindberg K. Rheinwald J. G. (1989). Suprabasal 40 kd keratin (K19) expression as an immunohistologic marker of premalignancy in oral epithelium. *Am. J. Pathol.* 134 89–98. 2464285 PMC1879556

[B33] Little H. J. Rorick N. K. Su L. I. Baldock C. Malhotra S. Jowitt T. (2009). Missense mutations that cause Van der Woude syndrome and popliteal pterygium syndrome affect the DNA-binding and transcriptional activation functions of IRF6. *Hum. Mol. Genet.* 18 535–545. 10.1093/hmg/ddn381 19036739 PMC2638798

[B34] Liu H. Leslie E. J. Jia Z. Smith T. Eshete M. Butali A. (2016). Irf6 directly regulates Klf17 in zebrafish periderm and Klf4 in murine oral epithelium, and dominant-negative KLF4 variants are present in patients with cleft lip and palate. *Hum. Mol. Genet.* 25 766–776. 10.1093/hmg/ddv614 26692521 PMC4743694

[B35] Maquat L. E. (2002). Nonsense-mediated mRNA decay. *Curr. Biol.* 12 R196–R197. 10.1016/S0960-9822(02)00747-9 11909543

[B36] Meng L. Bian Z. Torensma R. Von Den Hoff J. W. (2009). Biological mechanisms in palatogenesis and cleft palate. *J. Dent. Res.* 88 22–33. 10.1177/0022034508327868 19131313

[B37] Mossey P. A. Little J. Munger R. G. Dixon M. J. Shaw W. C. (2009). Cleft lip and palate. *Lancet* 374 1773–1785. 19747722 10.1016/S0140-6736(09)60695-4

[B38] Murray J. C. Nishimura D. Y. Buetow K. H. Ardinger H. H. Spence M. A. Sparkes R. S. (1990). Linkage of an autosomal dominant clefting syndrome (Van der Woude) to loci on chromosome Iq. *Am. J. Hum. Genet.* 46 486–491. 2309700 PMC1683619

[B39] Nagy E. Maquat L. E. (1998). A rule for termination-codon position within intron-containing genes: when nonsense affects RNA abundance. *Trends Biochem. Sci.* 23 198–199. 10.1016/S0968-0004(98)01208-0 9644970

[B40] Oberbeck N. Pham V. C. Webster J. D. Reja R. Huang C. S. Zhang Y. (2019). The RIPK4-IRF6 signalling axis safeguards epidermal differentiation and barrier function. *Nature* 574 249–253. 10.1038/s41586-019-1615-3 31578523

[B41] Osoegawa K. Vessere G. M. Utami K. H. Mansilla M. A. Johnson M. K. Riley B. M. (2008). Identification of novel candidate genes associated with cleft lip and palate using array comparative genomic hybridisation. *J. Med. Genet.* 45 81–86. 10.1136/jmg.2007.052191 17873121 PMC3732463

[B42] Parada-Sanchez M. T. Chu E. Y. Cox L. L. Undurty S. S. Standley J. M. Murray J. C. (2017). Disrupted IRF6-NME1/2 complexes as a cause of cleft lip/palate. *J. Dent. Res.* 96 1330–1338. 10.1177/0022034517723615 28767310 PMC5613882

[B43] Peyrard-Janvid M. Leslie E. J. Kousa Y. A. Smith T. L. Dunnwald M. Magnusson M. (2014). Dominant mutations in GRHL3 cause Van der Woude syndrome and disrupt oral periderm development. *Am. J. Hum. Genet.* 94 23–32. 10.1016/j.ajhg.2013.11.009 24360809 PMC3882735

[B44] Peyrard-Janvid M. Pegelow M. Koillinen H. Larsson C. Fransson I. Rautio J. (2005). Novel and de novo mutations of the IRF6 gene detected in patients with Van der Woude or popliteal pterygium syndrome. *Eur. J. Hum. Genet.* 13 1261–1267. 10.1038/sj.ejhg.5201493 16160700

[B45] Popp M. W. Maquat L. E. (2016). Leveraging rules of nonsense-mediated mRNA decay for genome engineering and personalized medicine. *Cell* 165 1319–1322. 10.1016/j.cell.2016.05.053 27259145 PMC4924582

[B46] Qin B. Y. Liu C. Lam S. S. Srinath H. Delston R. Correia J. J. (2003). Crystal structure of IRF-3 reveals mechanism of autoinhibition and virus-induced phosphoactivation. *Nat. Struct. Biol.* 10 913–921. 10.1038/nsb1002 14555996

[B47] Rhea L. Canady F. J. Le M. Reeb T. Canady J. W. Kacmarynski D. S. F. (2020). Interferon regulatory factor 6 is required for proper wound healing in vivo. *Dev. Dyn.* 249 509–522. 10.1002/dvdy.134 31724286 PMC9266192

[B48] Richards S. Aziz N. Bale S. Bick D. Das S. Gastier-Foster J. (2015). Standards and guidelines for the interpretation of sequence variants: a joint consensus recommendation of the American College of Medical Genetics and Genomics and the Association for Molecular Pathology. *Genet. Med.* 17 405–424. 10.1038/gim.2015.30 25741868 PMC4544753

[B49] Richardson R. J. Dixon J. Jiang R. Dixon M. J. (2009). Integration of IRF6 and Jagged2 signalling is essential for controlling palatal adhesion and fusion competence. *Hum. Mol. Genet.* 18 2632–2642. 10.1093/hmg/ddp201 19439425 PMC2701335

[B50] Richardson R. J. Dixon J. Malhotra S. Hardman M. J. Knowles L. Boot-Handford R. P. (2006). Irf6 is a key determinant of the keratinocyte proliferation-differentiation switch. *Nat. Genet.* 38 1329–1334. 10.1038/ng1894 17041603

[B51] Richardson R. J. Hammond N. L. Coulombe P. A. Saloranta C. Nousiainen H. O. Salonen R. (2014). Periderm prevents pathological epithelial adhesions during embryogenesis. *J. Clin. Invest.* 124 3891–3900. 10.1172/JCI71946 25133425 PMC4151209

[B52] Rizos M. Spyropoulos M. N. (2004). Van der Woude syndrome: a review. Cardinal signs, epidemiology, associated features, differential diagnosis, expressivity, genetic counselling and treatment. *Eur. J. Orthod.* 26 17–24. 10.1093/ejo/26.1.17 14994878

[B53] Schutte B. C. Basart A. M. Watanabe Y. Laffin J. J. Coppage K. Bjork B. C. (1999). Microdeletions at chromosome bands 1q32-q41 as a cause of Van der Woude syndrome. *Am. J. Med. Genet.* 84 145–150. 10.1002/(SICI)1096-8628(19990521)84:2<145::AID-AJMG11>3.0.CO;2-L 10323740

[B54] Schutte B. C. Bjork B. C. Coppage K. B. Malik M. I. Gregory S. G. Scott D. J. (2000). A preliminary gene map for the Van der Woude syndrome critical region derived from 900 kb of genomic sequence at 1q32-q41. *Genome Res.* 10 81–94. 10645953 PMC310500

[B55] Schweingruber C. Rufener S. C. Zund D. Yamashita A. Muhlemann O. (2013). Nonsense-mediated mRNA decay - mechanisms of substrate mRNA recognition and degradation in mammalian cells. *Biochim. Biophys. Acta* 1829 612–623. 10.1016/j.bbagrm.2013.02.005 23435113

[B56] Stenson P. D. Mort M. Ball E. V. Evans K. Hayden M. Heywood S. (2017). The Human Gene Mutation Database: towards a comprehensive repository of inherited mutation data for medical research, genetic diagnosis and next-generation sequencing studies. *Hum. Genet.* 136 665–677. 10.1007/s00439-017-1779-6 28349240 PMC5429360

[B57] Tamura T. Yanai H. Savitsky D. Taniguchi T. (2008). The IRF family transcription factors in immunity and oncogenesis. *Annu. Rev. Immunol.* 26 535–584. 10.1146/annurev.immunol.26.021607.090400 18303999

[B58] Van Beurden H. E. Von Den Hoff J. W. Torensma R. Maltha J. C. Kuijpers-Jagtman A. M. (2005). Myofibroblasts in palatal wound healing: prospects for the reduction of wound contraction after cleft palate repair. *J. Dent. Res.* 84 871–880. 10.1177/154405910508401002 16183784

[B59] Van Der Woude A. (1954). Fistula labii inferioris congenita and its association with cleft lip and palate. *Am. J. Hum. Genet.* 2 244–256. PMC1716548 13158329

[B60] Wehby G. L. Cassell C. H. (2010). The impact of orofacial clefts on quality of life and healthcare use and costs. *Oral. Dis.* 16 3–10. 10.1111/j.1601-0825.2009.01588.x 19656316 PMC2905869

[B61] Wilkie A. O. Morriss-Kay G. M. (2001). Genetics of craniofacial development and malformation. *Nat. Rev. Genet.* 2 458–468. 10.1038/35076601 11389462

[B62] Wilusz C. J. Wang W. Peltz S. W. (2001). Curbing the nonsense: the activation and regulation of mRNA surveillance. *Genes Dev.* 15 2781–2785. 11691829 10.1101/gad.943701

[B63] Zucchero T. M. Cooper M. E. Maher B. S. Daack-Hirsch S. Nepomuceno B. Ribeiro L. (2004). Interferon regulatory factor 6 (IRF6) gene variants and the risk of isolated cleft lip or palate. *N. Engl. J. Med.* 351 769–780. 10.1056/NEJMoa032909 15317890

