# Supplementary material for: A Novel Van der Woude Syndrome-Causing IRF6 Variant Is Subject to Incomplete Non-sense-Mediated mRNA Decay Affecting the Phenotype of Keratinocytes
Source: Front Cell Dev Biol. 2020 Sep 29;8:583115. doi: 10.3389/fcell.2020.583115 (PMC7552806; doi:10.3389/fcell.2020.583115)
Supplement: Supplementary file 1 [file Data_Sheet_1.PDF]

Supplementary Material

A novel Van der Woude syndrome-causing *IRF6* variant is subject to nonsense-mediated mRNA decay affecting the phenotype of keratinocytes

Martin Degen[1#*], Eleftheria Girousi[1#], Julia Feldmann[1], Ludovica Parisi[1], Giorgio C La Scala[2], Isabelle Schnyder[3], André Schaller[4], Christos Katsaros[1]

*** Correspondence:** Martin Degen
martin.degen@zmk.unibe.ch

1 Supplementary Figures and Tables

1.1 Supplementary Figures

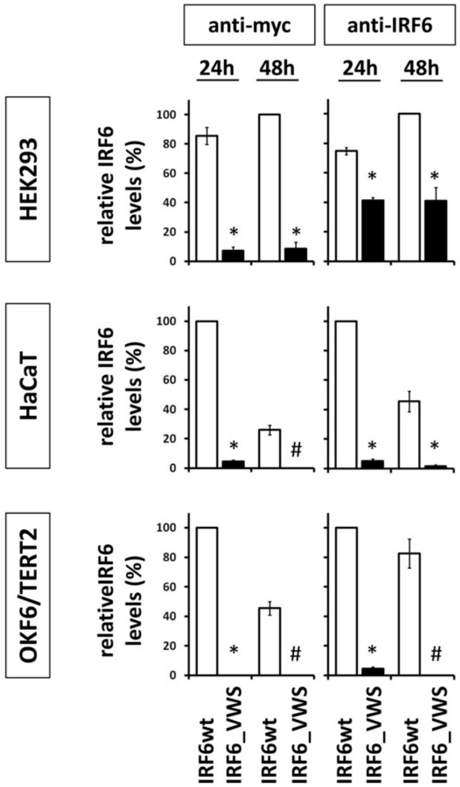

Supplementary Figure 1. Quantification of the relative IRF6 levels from the immunoblots in HEK293, HaCaT, and OKF6/TERT2 cells transfected with IRF6wt (white) and IRF6_VWS (black). * $p \leq 0.05$ vs. IRF6wt (24 h / 48 h); # not detectable.

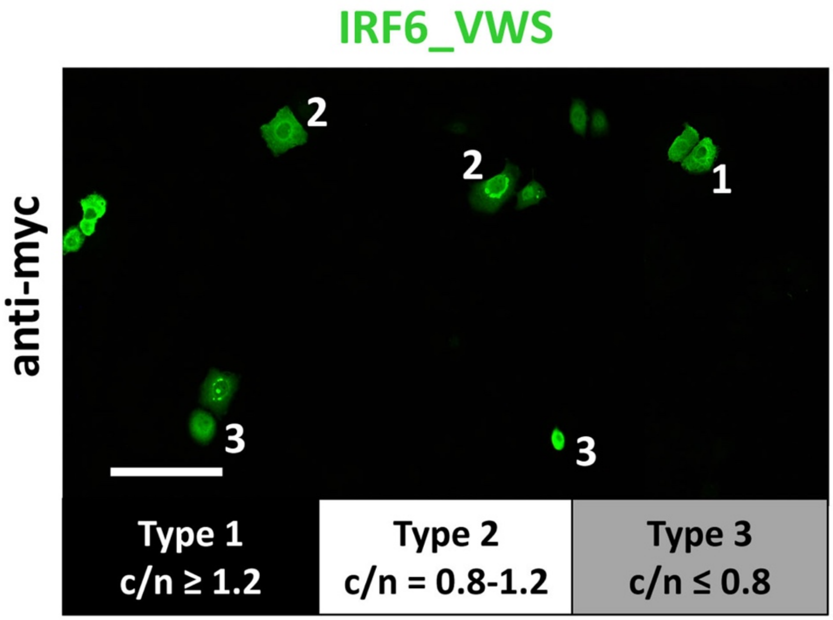

Supplementary Figure 2. A representative image of HaCaT cells transfected with the IRF6_VWS construct stained with the anti-myc antibody. Indicated are the three types of cellular IRF6_VWS localization as assessed by linescan plots. Scale bar: 50 µm

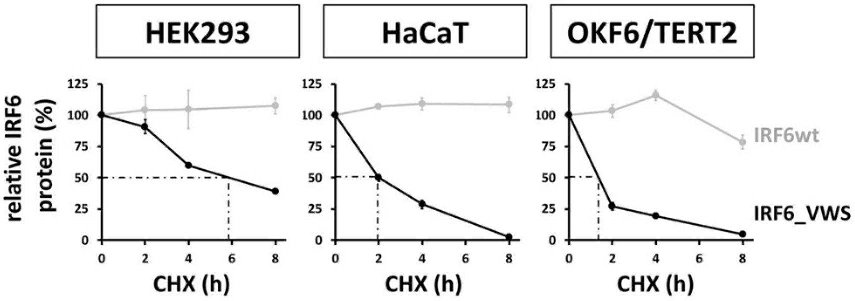

Supplementary Figure 3. Quantification (of anti-IRF6 immunoblots) of the relative IRF6wt (gray) and IRF6_VWS (black) levels in HEK293, HaCaT, and OKF6/TERT2 cells transfected with IRF6wt and IRF6_VWS, respectively, and treated with cycloheximide (CHX).

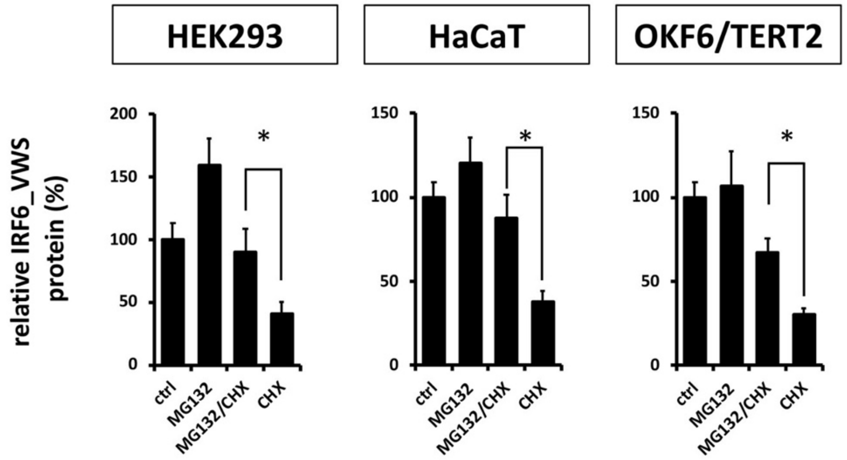

Supplementary Figure 4. Densitometric quantification of the relative IRF6_VWS levels in HEK293, HaCaT, and OKF6/TERT2 cells transfected with IRF6_VWS and treated with either MG132, CHX, or MG132/CHX (anti-IRF6 immunoblots). * $p \leq 0.05$.

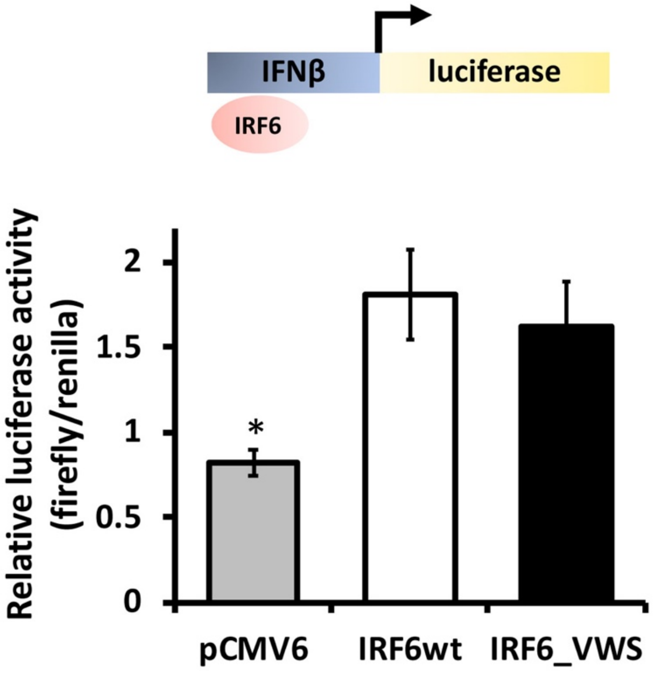

Supplementary Figure 5. HEK293T cells were transfected with an *IFNβ* promoter reporter plasmid together with plasmids expressing IRF6wt and IRF6_VWS. Gene reporter activity was measured 24 h post-transfection and is shown as the fold increase over cells transfected with empty plasmid. * $p \leq 0.05$ vs. IRF6wt, IRF6_VWS.

Figure 3D

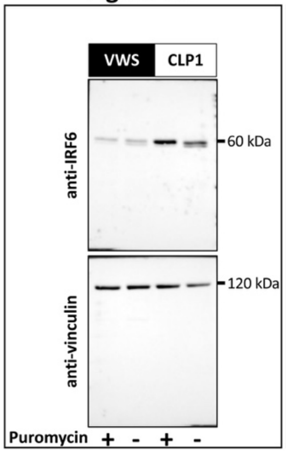

Figure 4C

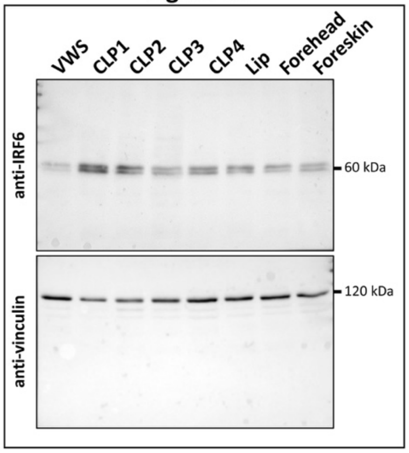

Figure 5C

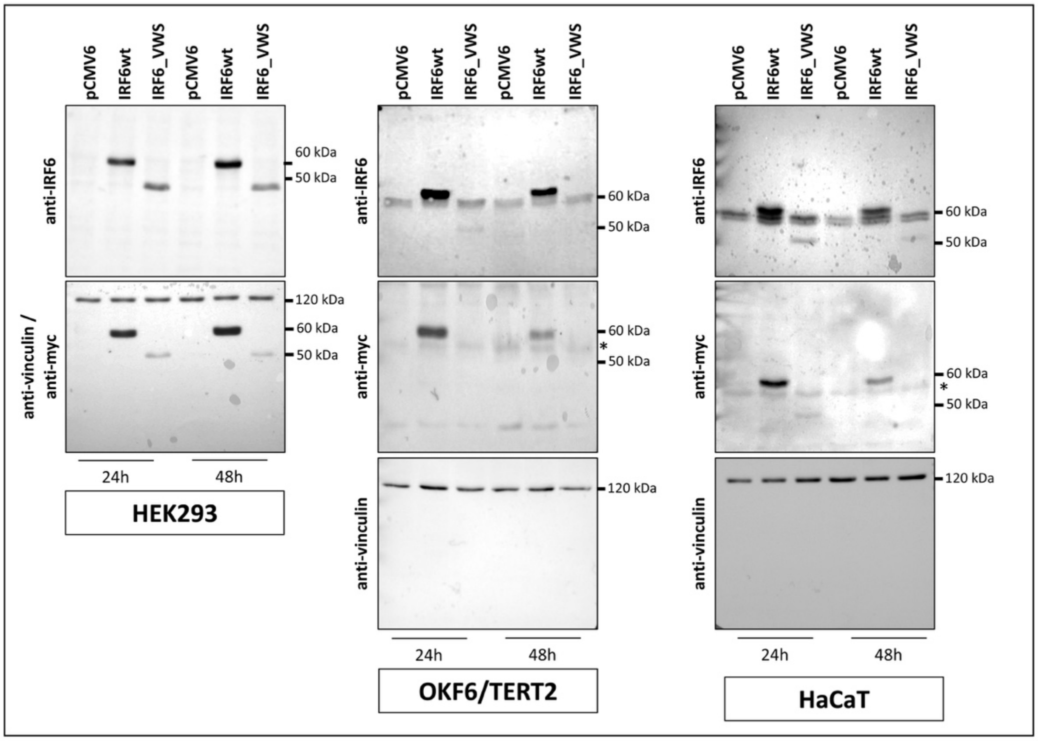

Figure 7A

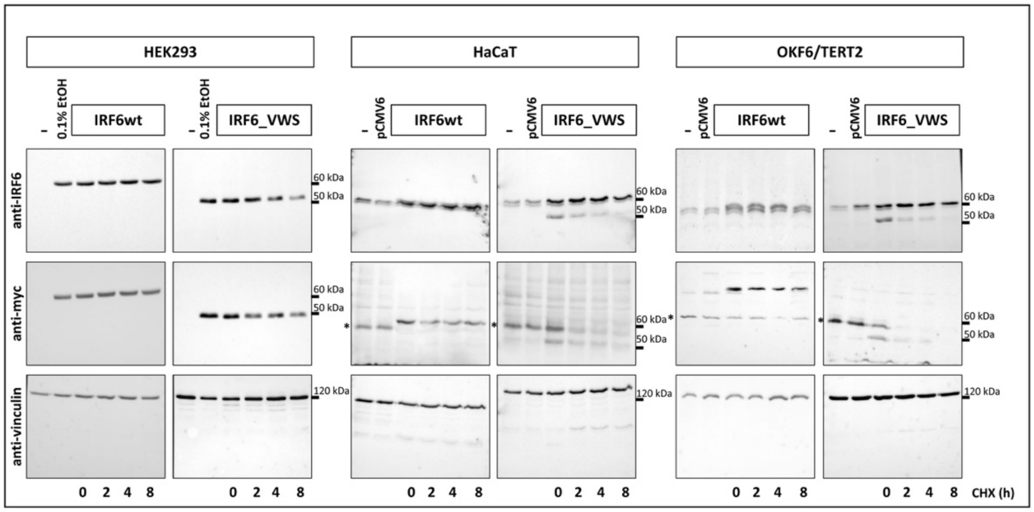

Figure 7C

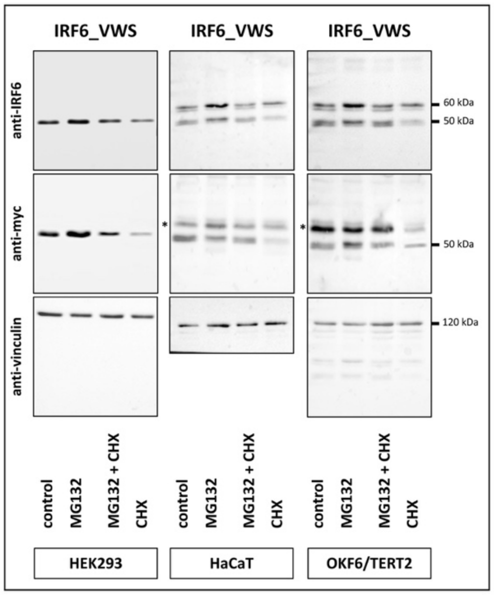

Figure 8C

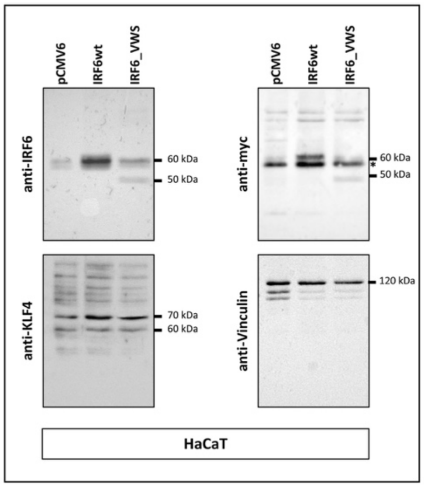

Supplementary Figure 6. Full-length blots of the immunoblot analyses of Figures 3, 4, 5, 7 and 8 are shown. * background bands.

1.2 Supplementary Tables

GENE	SPECIFICS	PRIMER SEQUENCE FORWARD (5'-3')	PRIMER SEQUENCE REVERSE (5'-3')	amplicon size (bp)	used in
Keratin 4 (KRT4)		CAACCTGAAGAACACCAAGA	AAGAGTCTGGCACTGCTTC	100	Fig. 1
Keratin 13 (KRT13)		CTGAACAAGGAGGTGTCTACCA	ATAGCGGCACTCCGTCTCT	162	Fig. 1
Keratin 14 (KRT14)		GGCCTGCTGAGATCAAAGACTAC	CACTGTGGCTGTGAGAATCTTGTT	80	Fig. 1
Keratin 19 (KRT19)		TGAGTGACATGCGAAGCCAAT	ACCTCCCGGTTCAATTCTTCA	100	Fig. 1
Loricrin (LOR)		AGACCCAGCAGAAGCAGGCG	AGCAGAACTAGATGCAGCCG	200	Figs. 1, 9
Filaggrin (FLG)		CTGGACACTCAGGTTCCCAT	TTTCGTGTTTGTCTGCTTGC	103	Figs. 1, 9
Interferon Regulatory Factor 6 (IRF6)	total *IRF6 endogenous*	GCTCTTCCATATCATGGCCCTC	CTACAGCCCAGGCCTTAAAAA	200	Figs. 4, 5
Interferon Regulatory Factor 6 (IRF6)	IRF6wt endogenous	GAGCAGGTCAAATTCCCAGG	GGCCCAGACCAGTACAC	164	Figs. 4, 5
Interferon Regulatory Factor 6 (IRF6)	*IRF6_VWS endogenous*	GAGCAGGTCAAATTCCCAGG	CCCAGACCAGGCTTGCAC	158	Figs. 4, 5
Interferon Regulatory Factor 6 (IRF6)	total *IRF6 endogenous and exogenous*	CAAGTGTGTGACATCCCTCAG	CCACATCATTATCCTTCTCATCC	91	Fig. 6
Interferon Regulatory Factor 6 (IRF6)	*IRF6wt exogenous*	CCTTCAAACCCAGGAGAGCTG	CTGCCAGATCCTCTTCTGAGAT	128	Fig. 6
Interferon Regulatory Factor 6 (IRF6)	*IRF6_VWS exogenous*	CAGCCTGGAGCAGGTCAAAT	CCCAGACCAGGCTTGCAC	165	Fig. 6
Interferon Regulatory Factor 6 (IRF6)	*total IRF6 endogenous*	CACTGCCTGCTCTTCCA	CTACAGCCCAGGCCTTAAAAA	208	Fig. 9
Grainyhead like transcription factor 3 (GRHL3)		CCCCCATGTCCAGAGGACTA	ACTCGCCTGACTTGATGTGG	91	Fig. 9
Krüppel like factor 4 (KLF4)		GAGAAGACACTGCGTCAAGC	AGTCGCTTCATGTGGGAGA	71	Fig. 9
Ovo Like Transcriptional Repressor 1 (OVOL1)		CCGTGCGTCTCCACGTGCAA	GGCTGTGGTGGGCAGAAGCC	101	Fig. 9
Glyceraldehyde 3-phosphate dehydrogenase (GAPDH)		CTCTGACTTCAACAGCGACACCC	TCCTCTTGTGCTCTTGCTGGGGC	199	

Supplementary Table 1. Sequence of the qPCR primers used in this study. bp: base pairs
